# Synthesis and *in Vitro* Antiproliferative Evaluation of Some B-norcholesteryl Benzimidazole and Benzothiazole Derivatives

**DOI:** 10.3390/md13042488

**Published:** 2015-04-22

**Authors:** Jianguo Cui, Binbin Qi, Chunfang Gan, Zhipin Liu, Hu Huang, Qifu Lin, Dandan Zhao, Yanmin Huang

**Affiliations:** 1 College of Chemistry and Material Science, Guangxi Teachers Education University, Nanning 530001, China; E-Mails: cuijg1954@126.com (J.C.); 554377663@qq.com (B.Q.); ganchunfang2008@126.com (C.G.); 573052962@qq.com (Z.L.); 404045774@qq.com (D.Z.); 2 Guangxi Key Laboratory of Beibu Gulf Marine Biodiversity Conservation, Qizhou University, Qizhou 535099, China; E-Mails: mrhuanghu@126.com (H.H.); linqifu335@163.com (Q.L.)

**Keywords:** cholesterol, B-norcholesteryl benzimidazoles, B-norcholesteryl benzothiazoles, antiproliferative activity, apoptosis

## Abstract

Taking orostanal (a compound from a Japanese marine sponge, *Stelletta hiwasaensis*) as a lead compound, some novel B-norcholesteryl benzimidazole and benzothiazole derivatives were synthesized. The antiproliferative activity of the compounds against human cervical carcinoma (HeLa), human lung carcinoma (A549), human liver carcinoma cells (HEPG2) and normal kidney epithelial cells (HEK293T) was assayed. The results revealed that the benzimidazole group was a better substituent than benzothiazole group for increasing the antiproliferative activity of compounds. 2-(3β′-Acetoxy-5β′-hydroxy-6′-B-norcholesteryl)benzimidazole (**9b**) with the structure of 6-benzimidazole displays the best antiproliferative activity to the cancer cells in all compounds, but is almost inactive to normal kidney epithelial cells (HEK293T). The assay of compound **9b** to cancer cell apoptosis by flow cytometry showed that the compound was able to effectively induce cancer cell apoptosis. The research provided a theoretical reference for the exploration of new anti-cancer agents and may be useful for the design of novel chemotherapeutic drugs.

## 1. Introduction

Cancer is the leading cause of death in economically developed countries and the second leading cause of death in developing countries [1]. WHO reported that the incidence of cancer will increase by 50%, and the global annual increase of the number of cancer patients will reach 15,000,000 in 2020. So, finding novel chemotherapeutic agents with excellent antiproliferative activity and high therapeutic compounds remain an important target for scientists [2].

**Figure 1:**
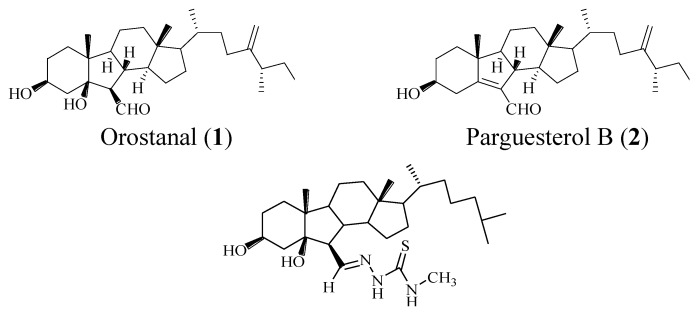
The structures of compounds **1**–**3**.

The discovery of new compounds from natural sources has been very important in pharmacologic science research. The past decade has witnessed an increase in the number of compounds from the screening of diverse marine invertebrates, such as soft corals, sponges and tunicates. In 2001, a novel sterol, named orostanal (**1**) (Figure 1), possessing a contracted cyclopentane B-ring was isolated from a Japanese marine sponge, *Stelletta hiwasaensis*. Orostanal induced apoptosis in HL-60 cells at 10 μg/mL, and inhibited 50% cell growth at 1.7 μM [3]. After that, another novel 5(6-7)abeo-sterol named parguesterol B (**2**) was obtained from the Caribbean Sea sponge *Svenzea zeai*, and it was a moderately strong anti-tuberculosis molecule with a MIC value of 7.8 μg/mL [4].

3β,5β-Dihydroxyl-6-(4′-methyl)-thiosemicarbazone-B-norcholestane (**3**)

Since compound **1** displays an excellent antiproliferative activity by inducing the apoptosis of tumor cell, it aroused our great interest to design and synthesize some steroidal compounds with 6,5,6,5 fused rings and assay their antiproliferative activity. In previous work, taking orostanal as a lead compound, we had prepared a series of analogs of compound **1** or **2** with different substituted groups and various side chains, and evaluated their antiproliferative activities [5,6]. The results showed that the presence of a cholesterol-type side chain was very important in determining the cytotoxicity of these compounds, and the presence of a thiosemicarbazone group at the C-6 position of steroid nucleus could enhance the antiproliferative activity of the compounds. For example, compound **3** exhibited excellent antiproliferative activities with an IC_50_ value of 13.8 and 5.4 μM against SGC-7901 (human ventriculi carcinoma) and HeLa (human cervical carcinoma) cells [7].

Heterosteroids have been accredited with a great amount of attention over the years by medicinal chemists for drug discovery. The incorporation of a heterocyclic ring or a heteroatom in the steroidal skeleton affects the chemical properties of a steroid and often results in useful alterations in its biological activities [8,9]. Some heterosteroids display an excellent anticancer activitiy, e.g., anticancer agents like 2-methoxyestradiol [10], exemestane [11], estramustine phosphate sodium [12] and abiraterone [13]. So far, the steroids containing heterocycles had been widely explored and reported [14]. Literatures suggested that such compounds displayed distinct cytotoxicity against cancer cell lines [15,16,17,18,19].

In order to obtain biologically potent anticancer compounds with diverse structures, as an extension of our previous work, a series of B-norsteroidal compounds possessing a 6,5,6,5 fused ring and the structure of 6-benzimidazole or 6-benzothiazole had been prepared starting from cholesterol in the present study. Meanwhile the antiproliferative activity of the compounds *in vitro* was evaluated further.

## 2. Results and Discussion

### 2.1. Chemistry

**Scheme 1:**
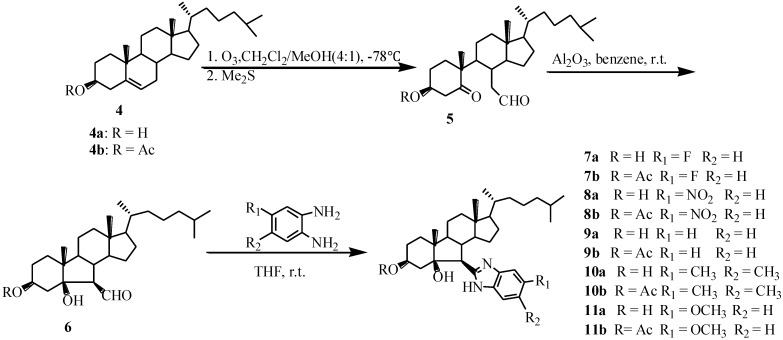
Synthesis of compounds **7**–**11**.

Scheme 1 outlines the synthetic procedure of B-norcholesteryl benzimidazole compounds (**7**–**11**). Compound **6** was prepared according to Gan, C.F. [5]. The configurations of C-5 and C-7 in compound **6** had been described in references [20,21,22]. The reaction of compound **6** with *O*-phenylenediamine afforded a corresponding benzimidazole group in the 6-position of steroidal nucleus [23]. To investigate the effect of various substituent groups in benzene ring on the antiproliferative activity of compounds, compounds (**7**–**11**) were synthesized by the reaction of **6** with different *O*-phenylenediamine derivatives. Their structures were confirmed on analytical and spectral data. In the NMR spectrum, resonances signals of Ar-H at 6.93, 7.22, 7.44 ppm and Ar-C at 160.7–110.0 ppm proved the formation of benzimidazole in compound **7a**.

**Scheme 2:**
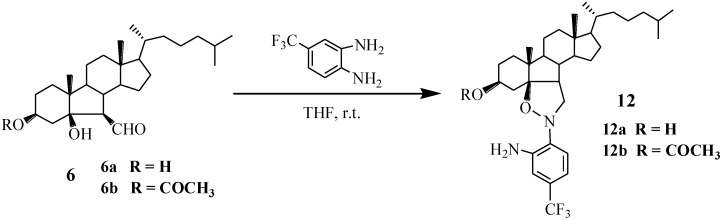
Synthesis of compounds **12**.

However, when compound **6** reacted with 4-trifluoromethyl-*O*-phenylenediamine, the anticipated compound, 2-(3β′,5β′-dihydroxy-6′-B-norcholesteryl)-5-trifluoromethylbenzimidazole was not produced. Interestingly, we obtained compound **12** possessing an isoxazolidine ring structure (Scheme 2). Because the nucleophilicity of 2′-NH_2_ is largely decreased due to a negative inductive effect of trifluoromethyl group, it cannot form the structure of benzimidazole, and compound **12** is generated. The structure of **12** was confirmed by analysis of ^1^H, ^13^C NMR, DEPT135/90 and HMQC (see Supplementary Information).

**Scheme 3:**
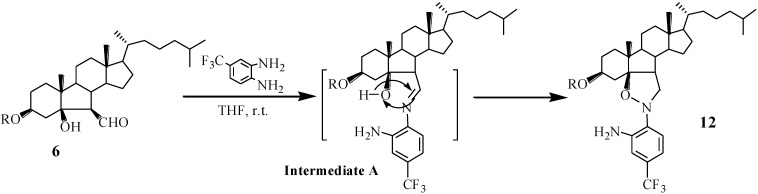
The mechanism of formation of compound **12**.

A proposed mechanism for the formation of compounds **12** is shown in Scheme 3. First, 1-NH_2_ of 4-trifluoromethyl-*O*-phenylenediamine attacks 6-aldehyde group of compound **6** to afford the intermediate A (Because of electron-withdrawing effect of trifluoromethyl, the nucleophilicity of 2-NH_2_ was decreased largely.). Subsequently, the coupling of imine with 5-hydroxyl in intermediate A gives compound **12** via migration of a hydrogen on hydroxyl.

**Scheme 4:**
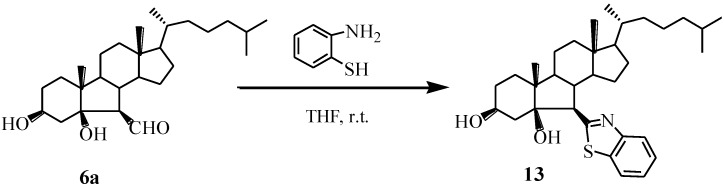
Synthesis of compound **13**.

In order to investigate the effect of S-containing benzimidazole on the antiproliferative activity, we prepared B-norcholesteryl benzothiazoles (**13**) by reacting compound **6a** with 2-aminothiophenol (Scheme 4). The structure of **13** was confirmed by analysis of IR, NMR and HRMS.

**Scheme 5:**
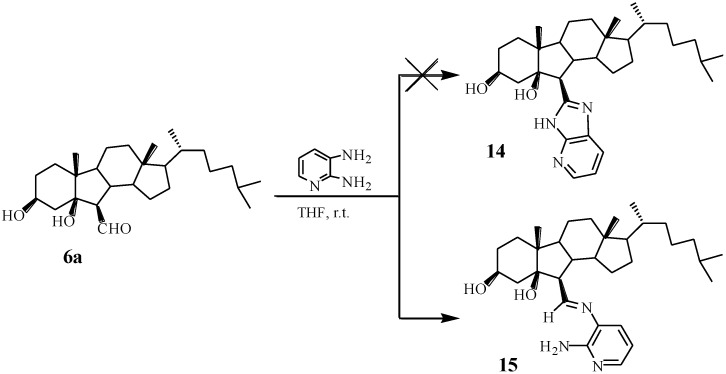
Synthesis of compound **15**.

Last, in order to determine the effect of benzene ring in benzimidazole on the cytotoxicity of compounds, we hoped to prepare compound **14** possessing a structure of pyridine ring but the anticipated compound **14** was not formed (Scheme 5). However, we obtained compound **15** with a structure of imine. The structure of **15** was confirmed by analysis of IR, NMR and HRMS.

### 2.2. Biological Results and Discussion

Lung cancer and liver cancer are a main cause of death in cancer patients. To evaluate the antiproliferative activity of the new compounds, we determined their IC_50_ values on A549 (human lung carcinoma), HEPG2 (human liver carcinoma) and HeLa (human cervical carcinoma) using a MTT assay, and non-cancer cells HEK293T (Normal Kidney Epithelial Cells) were chosen as a control. MTT is a compound that can be taken up by viable cells and reduced by a mitochondrial dehydrogenase forming a formazan product in living cells. The absorbance of the formazan at 492 nm is in linear proportion to cell numbers. The results were summarized as IC_50_ values (concentration of a compound allowing survival of 50% of the cells in a population) in µmol/L in Table 1.

**Table 1:** *In vitro* antiproliferative activities (IC_50_ in μmol/L) of compounds **7**–**15**.

Compounds	Cells			
	HeLa	A549	HEPG2	HEK293T
**7a**	4.2	66.7	>40	19.1
**7b**	15.9	28.2	21.2	20.3
**8a**	3.6	47.2	21.8	37.2
**8b**	31.2	50.6	30.3	>80
**9a**	4.9	61.2	29.6	53.3
**9b**	4.7	11.9	4.2	>80
**10a**	7.5	13.1	4.5	>80
**10b**	2.2	31.2	>40	>80
**11a**	7.5	14.0	8.4	>80
**11b**	11.8	>80	>40	>80
**12a**	16.6	27.0	12.2	>100
**12b**	3.7	>80	>80	>100
**13**	22.0	>80	>80	58.3
**15**	20.6	>80	>80	>80

As showed in Table 1, B-norcholesteryl benzimidazole derivatives (**7**–**11**) exhibited an excellent antiproliferative activity against HeLa cells. Except for compounds **7b**, **8b** and **11b**, the IC_50_ values of all compounds are under 10 µM. Thereinto, the most active compounds were **7a**, **8a**, **9a**, **9b** and **10a** (IC_50_ values: 4.2, 3.6, 4.9, 4.7 and 2.2, respectively). Obviously, compound **9b** with the structure of 6-benzimidazole displayed the best antiproliferative activity against A549 and HEPG2 cells while compound **10b** showed the best activity against HeLa cells in all compounds. Among all compounds synthesized, compound **9b** with benzimidazole, **10a** and **11a** with electron-donating groups in the benzimidazole displayed excellent cytotoxicity for HEPG2 cells (IC_50_: 4.2, 4.5 and 8.4, respectively).

After an electron-withdrawing group was introduced into the skeleton of benzimidazole, the cytotoxicity of the compounds **7b**–**8b** possessing 5-fluoro and 5-nitro resulted in slightly decreased. However, compounds **10a**, **10b** and **11a** with electron-donating groups in the benzene ring showed a similar cytotoxicity compare with compounds **9a** and **9b**. Interestingly, except compound **9a**, compound **9b** and compounds **10**–**11** with the electron-donating groups were almost inactive to normal kidney epithelial cells (HEK293T), but compounds **7** and **8a** with the electron-withdrawing groups showed distinct cytotoxicity to same kind of cells.

After the 3-hydroxyl group on **8a** was transformed into 3-acetoxyl, the antiproliferative activity of compound **8b** against these cells decreased greatly, suggesting the importance of the hydroxyl group in the compound **8**. However, after the 3-hydroxyl group in **9a** was transformed into 3-acetoxyl, the antiproliferative activity of forming compound **9b** obtained a prominent increase against the cancer cells and an obvious decrease on normal kidney epithelial cells. These results demonstrated that compound **9b** based on the 3-acetoxyl and 6-benzimidazole was a potent antiproliferative agent.

Compound **12a** bearing an isoxazolidine ring structure displayed also distinct cytotoxicity against these cancer cells. However, compound **12b** showed an excellent selective cytotoxicity against HeLa cells with an IC_50_ value of 3.7 µM, and was almost inactive on other cancer cells. Both of **12a** and **12b** were inactive on normal kidney epithelial cells.

Apparently, after the N atom of benzimidazole was substituted by S atom, the cytotoxic activity of the forming compound **13** was markedly decreased (compare **9a** and **13**). The results demonstrate that benzimidazole group is a better substituent than benzothiazole group for increasing the antiproliferative activity of compounds and suggest that the analogs based on the 6-benzimidazole scaffold may constitute a novel class of antiproliferative agents, which deserve further study. Obviously, compound **15** with the structure of pyridine ring didn’t display distinct cytotoxicity against all these cells except HeLa cells.

The Selectivity Index (SI) was defined as the ratio of the cytotoxicity of a compound with respect to normal cells (IC_50_ HEK293T) *versus* cancer cells and used to determine the criterion of effectiveness of the compounds. The SI values of the compounds are listed in Table 2.

One important criterion for a therapeutic drug for cancer is to have minimal or no side effects to normal body cells of patients undergoing chemotherapy. Taking into account that a higher SI corresponds to greater overall anticancer activity, we can identify the leading compounds as **9b**, **10a** and **11a** (SI values for HeLa cells: 17.0, 10.7, 10.7; A549 cells: 6.7, 6.1, 5.7; HEPG2: 19.0, 17.8, 9.5). Meanwhile, compound **10b** displayed the best excellent selective inhibition against HeLa cells, and the SI was 36.4. So, comparison of the cytotoxicity of the compounds with the SI values suggested that the compounds **9b**, **10a** and **11a** may be potent anticancer agents and compounds **10b** and **12b** are excellent inhibitors against human cervical carcinoma (HeLa), which deserve further study.

**Table 2:** SI values of the compounds **7**–**15**.

Compounds	SI _HeLa_	SI _A549_	SI _HEPG2_
**7a**	4.5	-	-
**7b**	1.3	-	-
**8a**	10.3	-	1.7
**8b**	2.6	1.6	2.6
**9a**	10.9	-	1.8
**9b**	17.0	6.7	19.0
**10a**	10.7	6.1	17.8
**10b**	36.4	2.6	-
**11a**	10.7	5.7	9.5
**11b**	6.8	-	-
**12a**	6.0	3.7	8.2
**12b**	27.0	-	-
**13**	2.7	-	-
**15**	3.9	-	-

A comparison of the structures of the synthesized compounds with pronounced biological activity makes it possible to identify some structure/biological activity relationships for these B-norcholesteryl benzimidazole and benzothiazole derivatives:

(1) The 6-benzimidazole group is a better substituent than 6-benzothiazole group for increasing the antiproliferative activity of compounds (compare **9a** and **13**).
(2) The presence of electron-withdrawing groups in the benzimidazole will decrease the cytotoxicity of the compounds and electron-donating groups show not an obvious effect for cytotoxicity of compounds (compare **7b**, **8b** and **9b** or **9a**, **10a** and **11a**).
(3) Introduction of an isoxazolidine ring joined with ring B or an 6-imine moiety cannot increased the cytotoxicity of the compounds (compare **7a** and **12a**, **12b**). The introduction of pyridine ring in benzimidazole deceases the cytotoxicity of the compound on HeLa and HEPG2 cells (compare **9a**, **12a** and **15**).

**Figure 2:**
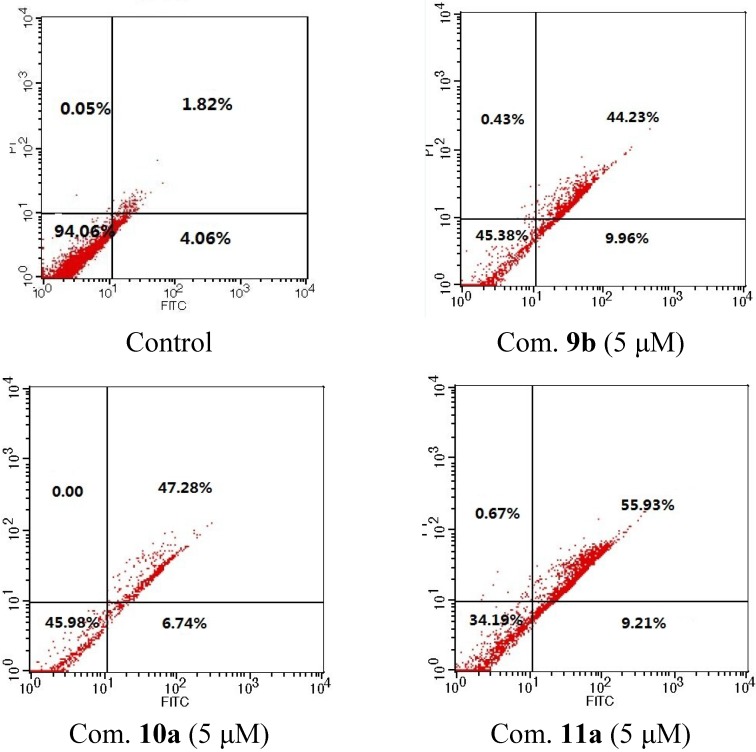
HeLa cells were double-stained with annexin V/PI and analyzed by flow cytometry. Treatment with compounds **9b**, **10a** and **11a** (5 μM) for 24 h induced apoptosis of HeLa cells.

To further disclose the molecular mechanism by which the compounds inhibit cancer cell proliferation, the HeLa cells were treated with compounds **9b**, **10a** and **11a**, and Annexin V assay was performed. The translocation of membrane phospholipid phosphatidylserine (PS) from the inner to the outer leaflet of the plasma membrane is an early event of cell apoptosis. Annexin V is a 35–36 kD Ca^2+^ dependent, phospholipid-binding protein that has a high affinity for PS. Therefore, FITC-conjugated Annexin V is commonly used to determine apoptotic cells at an early stage. As shown in Figure 2, treatment with 5 μM of **9b**, **10a** and **11a** resulted in 54.2%, 54.0% and 65.1% PI/Annexin V double-labeled apoptotic cells after 24 h incubation (the lower right quadrant and the upper right quadrant which contains early and late apoptotic cells, respectively), and necrotic cells are only 0.43%, 0.36% and 0.67% (the upper left quadrant), suggesting these compounds are potent apoptotic inducers in cervical carcinoma cells. There into, compound **11a** is more potent in induction of apoptosis in HeLa cells (Compare **11a** with **9b** and **10a**).

**Figure 3:**
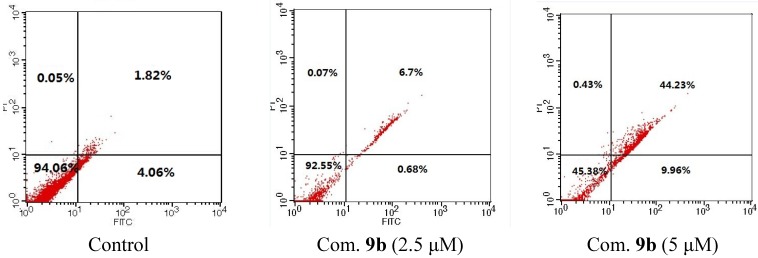
Dose depended apoptosis induced by compound **9b** for 24 h.

**Figure 4:**
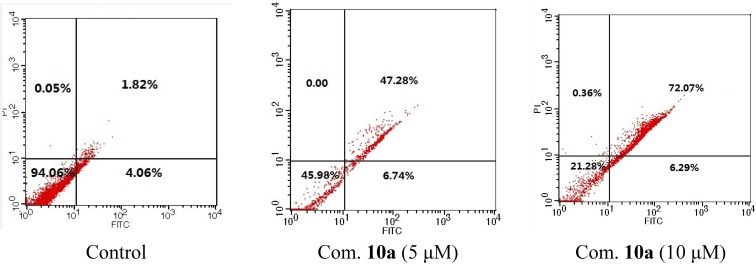
Dose depended apoptosis induced by compound **10a** for 24 h.

Similar results were observed after HeLa cells were treated with compounds **9b** and **10a** in a dose dependent manner (Figure 3 and Figure 4). Treatment with 5 μM of **9b** for 24 h resulted in 54.2% PI/Annexin V double-labeled apoptotic cells while **10a** could produce 78.4% in 10 μM condition.

## 3. Experimental Section

### 3.1. Chemistry

The sterols were purchased from Sinopharm Chemical Reagent Co., Ltd, Shanghai, China. All chemicals and solvents were analytical grade. Melting points were determined on an X_4_ apparatus (Beijing Tech Instrument Co. Ltd., Beijing, China) and were uncorrected. Infrared spectra were measured with a Thermo Scientific Nicolet IS-10 Spectrophotometer (Thermo Fisher Scientific, New York, NY, USA). The ^1^H and ^13^C NMR spectra were recorded in CDCl_3_ on a Bruker AV-600 spectrometer at working frequencies 600 and 150 MHz, and a Bruker AV-300 spectrometer at working frequencies 300 and 75 MHz, respectively. Chemical shifts are expressed in parts per million (δ) values and coupling constants (*J*) in Hertz. HREIMS was measured on an Agilent 6210 TOFMS instrument (Agilent Technologies, Palo Alto, CA, USA). The cell proliferation assay was undertaken by a MTT method using 96-well plates on a MLLTISKAN MK3 analysis spectrometer (Thermo Scientific, Shanghai, China). Annexin V assay was performed using FACS Calibur flow cytometry (Becton Dickinson, Biosciences, Franklin Lakes, NJ, USA).

Compound **6** was prepared according to the method in reference [5].

#### 3.1.1. General Procedure for the Synthesis of Compounds **7**–**11**

*O*-Phenylenediamine derivative (1.6 mmol) was added to a solution of compound **6** (1.0 mmol) in THF (50 mL). The solution was stirred for 8–24 h at room temperature until no starting material was observed (the progress of the reaction was monitored by TLC, petroleum ether/ethyl acetate = 2:1). Then the reaction was stopped and the majority of solvent was evaporated under reduced pressure. The residue was purified by flash chromatography on silica gel (300–400 mesh) to afford the corresponding target products **7**–**11**.

2-(3β′,5β′-Dihydroxy-6′-B-norcholesteryl)-5-fluorobenzimidazole (**7a**) White solid, yield: 45%, m. p. 139–140 °C. IR (KBr) ν/cm^−1^: 3252, 2945, 1626, 1599, 1524, 1444, 1382, 1142; ^1^H NMR (CDCl_3_, 300 MHz) δ: 0.64 (3H, s, 18′-CH_3_), 0.85 (6H, d, *J* = 6.6, 26′- and 27′-CH_3_), 0.91 (3H, d, *J* = 6.3, 21′-CH_3_), 1.06 (3H, s, 19′-CH_3_), 2.16–2.04 (2H, m, C8′-H and C4′-H), 2.70 (1H, d, *J* = 10.8, C7′-H), 3.89 (1H, br s, C3′-H), 6.93 (1H, td, *J* = 9.6, 2.4, 6-ArH), 7.22 (1H, d, *J* = 8.7, 7-ArH), 7.44 (1H, dd, *J* = 8.4, 4.5, 4-ArH); ^13^C NMR (CDCl_3_, 75 MHz) δ: 160.7 (2-C), 157.6 (5-C), 156.8 (8-C and 9-C), 110.3 (6-C and 7-C), 110.0 (4-C), 82.3 (5′-C), 66.9 (3′-C), 56.1 (17′-C), 55.4 (14′-C), 55.0 (7′-C), 54.6 (9′-C), 45.3 (13′-C), 45.2 (12′-C), 44.5 (24′-C), 42.3 (10′-C), 39.6 (8′-C), 39.4 (4′-C), 36.2 (22′-C), 35.5 (20′-C), 31.6 (1′-C), 28.4 (16′-C), 28.3 (25′-C), 28.0 (15′-C), 24.0 (2′-C), 23.8 (23′-C), 22.8 (26-C), 22.5 (27-C), 21.7 (11-C), 18.8 (21-C), 17.6 (19-C), 12.4 (18-C); HREIMS *m/z*: 525.3843 [M + H]^+^ (calcd. for C_33_H_50_FN_2_O_2_, 525.3856).

2-(3β′-Acetoxy-5β′-hydroxy-6′-B-norcholesteryl)-5-fluorobenzimidazole (**7b**) Yellow oil, yield: 69%. IR (KBr) ν/cm^−1^: 3274, 2942, 1729, 1534, 1464, 1237, 1018; ^1^H NMR (CDCl_3_, 600 MHz) δ: 0.56 (3H, s, 18′-CH_3_), 0.83 (6H, d, *J* = 4.2, 26′- or 27′-CH_3_), 0.89 (3H, d, *J* = 5.4, 21′-CH_3_), 1.07 (3H, s, 19′-CH_3_), 1.74 (3H, s, COCH_3_), 2.28 (1H, q, *J* = 10.8, C8′-H), 2.95 (1H, d, *J* = 11.4, C7′-H), 4.88 (1H, m, C3′-H), 6.89 (1H, t, *J* = 8.4, 6-ArH), 7.23 (1H, d, *J* = 7.8, 7-ArH), 7.44 (1H, s, 4-ArH); ^13^C NMR (CDCl_3_, 150 MHz) δ: 170.8 (3′-C=O), 160.8 (2-C), 157.7 (5-C), 155.9 (8-C and 9-C), 110.3 (6-C and 7-C), 109.9 (4-C), 81.4 (5′-C), 69.7 (3′-C), 55.7 (17′-C), 55.5 (14′-C), 54.3 (9′-C), 44.7 (12′-C), 44.4 (13′-C), 44.3 (10′-C), 39.8 (24′-C), 39.4 (8′-C), 38.6 (4′-C), 36.2 (22′-C), 35.4 (20′-C), 32.8 (1′-C), 28.5 (16′-C), 28.0 (25′-C), 25.3 (15′-C), 24.2 (2′-C), 23.7 (23′-C), 22.8 (26′-C), 22.5 (27′-C), 21.9 (COCH_3_), 21.0 (11′-C), 18.8 (21′-C), 18.4 (19′-C), 12.4 (18′-C); HREIMS *m/z*: 567.3951 [M + H]^+^ (calcd. for C_35_H_52_FN_2_O_3_, 567.3962).

2-(3β′,5β′-Dihydroxy-6′-B-norcholesteryl)-5-nitrobenzimidazole (**8a**) Yellow oil, yield: 78%. IR (KBr) ν/cm^−1^: 3369, 2935, 1587, 1517, 1462, 1317, 1165, 1070; ^1^H NMR (CDCl_3_, 300 MHz) δ: 0.65 (3H, s, 18′-CH_3_), 0.87 (6H, d, *J* = 6.3, 26′- and 27′-CH_3_), 0.93 (3H, d, *J* = 6.3, 21′-CH_3_), 1.09 (3H, s, 19′-CH_3_), 2.16-2.05 (1H, m, C8′-H and C4′-H), 2.72 (1H, d, *J* = 10.5, C7′-H), 4.00-3.90 (1H, m, C3′-H), 6.95 (1H, td, *J* = 9.6, 2.1, 7-ArH), 7.24 (1H, dd, *J* = 7.8, 6-ArH), 7.45 (1H, s, 4-ArH); ^13^C NMR (CDCl_3_, 75 MHz) δ: 160.8 (2-C), 157.7 (5-C), 156.8 (8-C), 131.0 (9-C), 128.9 (6-C), 110.4 (7-C), 110.1 (4-C), 82.3 (5′-C), 67.0 (3′-C), 56.1 (17′-C), 55.4 (14′-C), 54.9 (7′-C), 54.4 (9′-C), 45.6 (13′-C), 45.3 (12′-C), 44.6 (24′-C), 42.4 (10′-C), 39.6 (8′-C), 39.4 (4′-C), 36.2 (22′-C), 35.5 (20′-C), 31.3 (1′-C), 29.7 (16′-C), 28.4 (25′-C), 28.0 (15′-C), 24.2 (2′-C), 23.8 (23′-C), 22.8 (26′-C), 22.5 (27′-C), 21.7 (11′-C), 18.8 (21′-C), 17.7 (18′-C), 12.5 (19′-C); HREIMS *m/z*: 552.3805 [M + H]^+^ (calcd. for C_33_H_50_N_3_O_4_, 552.3801).

2-(3β′-Acetoxy-5β′-hydroxy-6′-B-norcholesteryl)-5-nitrobenzimidazole (**8b**) Yellow oil, yield: 41%. IR (KBr) ν/cm^−1^: 3428, 2945, 1706, 1615, 1517, 1467, 1337, 1253, 1068, 1018; ^1^H NMR (CDCl_3_, 600 MHz) δ: 0.63 (3H, s, 18′-CH_3_), 0.80 (3H, d, *J* = 6.6, 26′- or 27′-CH_3_), 0.81 (3H, d, *J* = 6.6, 26′- or 27′-CH_3_), 0.87 (3H, d, *J* = 6.6, 21′-CH_3_), 1.06 (3H, s, 19′-CH_3_), 1.93 (3H, s, 3′-COCH_3_), 2.26 (1H, q, *J* = 11.4, C8′-H), 2.88 (1H, d, *J* = 11.4, C7′-H), 3.53 (1H, br s, -OH), 5.04-5.00 (1H, m, C3′-H), 7.53 (1H, br s, 7-ArH), 8.10 (1H, dd, *J* = 8.4, 1.8, 6-ArH), 8.45 (1H, br s, 4-ArH); ^13^C NMR (CDCl_3_, 75 MHz) δ: 170.6 (3′-C=O ), 168.2 (2-C), 143.3 (5-C), 143.2 (8-C), 133.3 (9-C), 118.1 (6-C), 110.5 (7-C), 109.7 (4-C), 81.7 (5′-C), 69.9 (3′-C), 55.6 (17′-C), 55.4 (14′-C), 54.7 (7′-C), 54.6 (9′-C), 45.2 (13′-C), 45.1 (12′-C), 44.5 (24′-C), 39.6 (10′-C), 39.4 (8′-C), 39.3 (4′-C), 36.1 (22′-C), 35.5 (20′-C), 32.6 (1′-C), 28.4 (16′-C), 28.3 (25′-C), 28.0 (15′-C), 25.3 (2′-C), 23.8 (23′-C), 22.8 (26′-C), 22.5 (27′-C), 21.9 (CH_3_CO), 21.3 (11′-C), 18.6 (21′-C), 18.0 (18′-C), 12.5 (19′-C); HREIMS *m/z*: 594.3895 [M + H]^+^ (calcd. for C_35_H_52_N_3_O_5_, 594.3907).

2-(3β′,5β′-Dihydroxy-6′-B-norcholesteryl)benzimidazole (**9a**) Colourless oil, yield: 40%; IR (KBr) ν/cm^−1^: 3385, 2946, 2863, 1615, 1524, 1463, 1415, 1353, 1063, 1021; ^1^H NMR (CDCl_3_, 300 MHz) δ: 0.65 (3H, s, 18′-CH_3_), 0.86 (1H, d, *J* = 6.6, 26′- and 27′-CH_3_), 0.93 (1H, d, *J* = 6.3, 21′-CH_3_), 1.13 (1H, s, 19′-CH_3_), 2.77 (1H, d, *J* = 10.5, C7′-H), 4.07-3.99 (1H, m, C3′-H), 7.24 (2H, dd, *J* = 6.0, 3.0, 5-ArH and 6-ArH), 7.59 (2H, br s, 4-ArH and 7-ArH); ^13^C NMR (CDCl_3_, 75 MHz) δ: 169.2 (2-C), 155.4 (8-C and 9-C), 122.4 (5-C and 6-C), 122.2 (4-C and 7-C), 82.3 (5′-C), 67.1 (3′-C), 56.2 (17′-C), 55.5 (14′-C), 54.5 (7′-C), 53.9 (9′-C), 45.7 (13′-C), 45.3 (12′-C), 44.7 (24′-C), 42.8 (10′-C), 39.7 (8′-C), 39.4 (4′-C), 36.2 (22′-C), 35.6 (20′-C), 30.7 (1′-C), 29.7 (16′-C), 28.5 (25′-C), 28.0 (15′-C), 23.8 (2′-C), 22.8 (23′-C), 22.7 (26′-C), 22.6 (27′-C), 21.7 (11′-C), 18.8 (21′-C), 17.9 (19′-C), 12.5 (18′-C); HREIMS *m/z*: 507.3965 [M + H]^+^ (calcd. for C_33_H_51_N_2_O_2_, 507.3951).

2-(3β′-Acetoxy-5β′-hydroxy-6′-B-norcholesteryl)benzimidazole (**9b**) White solid, yield: 63%, m.p.: 160–162 °C. IR (KBr) ν/cm^−1^: 3451, 2945, 1714, 1614, 1526, 1454, 1362, 1265, 1023, 1023, 958; ^1^H NMR (CDCl_3_, 600 MHz) δ: 0.63 (3H, s, 18′-CH_3_), 0.83 (1H, d, *J* = 6.6, 26′- or 27′-CH_3_), 0.84 (1H, d, *J* = 6.6, 26′- or 27′-CH_3_), 0.89 (1H, d, *J* = 6.3, 21′-CH_3_), 1.08 (1H, s, 19′-CH_3_), 1.93 (1H, s, 3′-CH_3_CO), 2.06 (1H, d, *J* = 13.2, C4′-H), 2.21 (1H, q, *J* = 10.8, C8′-H), 2.82 (1H, d, *J* = 11.4, C7′-H), 5.01–4.95 (1H, m, C3′-H), 7.21–7.19 (2H, m, 5-ArH and 6-ArH), 7.55 (2H, br s, 4-ArH and 7-ArH); ^13^C NMR (CDCl_3_, 75 MHz) δ: 170.9 (3′-C=O), 170.6 (2-C), 154.7 (8-C and 9-C), 122.4 (5-C and 6-C), 122.1 (4-C and 7-C), 81.3 (5′-C), 70.0 (3′-C), 55.8 (17′-C), 55.4 (14′-C), 54.9 (7′-C), 54.4 (9′-C), 45.1 (13′-C), 44.9 (12′-C), 44.5 (24′-C), 39.7 (10′-C), 39.4 (8′-C), 39.3 (4′-C), 36.2 (22′-C), 35.5 (20′-C), 32.9 (1′-C), 28.5 (16′-C), 28.0 (25′-C), 25.4 (15′-C), 23.9 (2′-C), 23.8 (23′-C), 22.8 (26′-C), 22.5 (27′-C), 21.9 (CH_3_CO), 21.4 (11′-C), 18.8 (21′-C), 18.1 (19′-C), 12.5 (18′-C); HREIMS *m/z*: 549.4054 [M + H]^+^ (calcd. for C_35_H_53_N_2_O_3_, 549.4056).

2-(3β′,5β′-Dihydroxy-6′-B-norcholesteryl)-5, 6-dimethylbenzimidazole (**10a**) Brownish oil, yield: 77%. IR (KBr) ν/cm^−1^: 3429, 2945, 1634, 1534, 1464, 1377, 1232, 1018; ^1^H NMR (CDCl_3_, 600 MHz) δ: 0.63 (3H, s, 18′-CH_3_), 0.85 (3H, d, *J* = 6.6, 26′- or 27′-CH_3_), 0.86 (3H, d, *J* = 6.6, 26′- or 27′-CH_3_), 0.91 (3H, d, *J* = 6.0, 21′-CH_3_), 1.08 (3H, s, 19′-CH_3_), 2.11-2.04 (2H, m, C4′-H and C8′-H), 2.34 (6H, s, 5-CH_3_ and 6-CH_3_), 2.71 (1H, d, *J* = 10.8, C7′-H), 3.86 (1H, br s, C_3_-H), 7.34 (2H, br s, 4-ArH and 7-ArH); ^13^C NMR (CDCl_3_, 150 MHz) δ: 154.8 (2-C), 132.3 (5-C), 130.9 (6-C), 128.0 (8-C and 9-C), 118.6 (4-C and 7-C), 82.2 (5′-C), 67.0 (3′-C), 56.3 (17′-C), 55.5 (14′-C), 54.7 (7′-C), 54.4 (9′-C), 45.6 (13′-C), 45.2 (12′-C), 44.6 (24′-C), 42.6 (10′-C), 39.7 (8′-C), 39.5 (4′-C), 36.2 (22′-C), 35.5 (20′-C), 31.5 (1′-C), 28.5 (2′-C), 28.0 (16′-C), 24.2 (25′-C), 23.8 (15′-C), 22.8 (26′-C), 22.5 (27′-C), 21.7 (23′-C), 20.3 (11′-C), 18.9 (5-CH_3_ and 6-CH_3_), 18.8 (21′-C), 17.7 (19′-C), 12.5 (18′-C ); HREIMS *m/z*: 535.4270 [M + H]^+^ (calcd. for C_35_H_55_N_2_O_2_, 535.4264).

2-(3β′-Acetoxy-5β′-hydroxy-6′-B-norcholesteryl)-5, 6-dimethylbenzimidazole (**10b**) White solid, yield: 63%, m.p.: 218–219 °C. IR (KBr) ν/cm^−1^: 3274, 2942, 1729, 1534, 1465, 1359, 1237, 1018; ^1^H NMR (CD_3_OD, 300 MHz) δ: 0.74 (3H, s, 18′-CH_3_), 0.86 (6H, d, *J* = 6.6, 26′- and 27′-CH_3_), 0.94 (3H, d, *J* = 6.3, 21′-CH_3_), 1.07 (3H, s, 19′-CH_3_), 1.96 (1H, s, 3′-CH_3_CO), 2.12–2.03 (2H, m, C4′-H and C8′-H), 2.35 (6H, s, 5-CH_3_ and 6-CH_3_), 2.80 (1H, d, *J* = 11.7, C7′-H), 5.14–5.05 (1H, m, C_3_-H), 7.29 (2H, s, 4-ArH and 7-ArH); ^13^C NMR (CD_3_OD, 150 MHz) δ: 171.1 (3′-C=O), 153.6 (2-C), 130.4 (5-C, 6-C, 8-C and 9-C), 114.0 (4-C and 7-C), 80.8 (5′-C), 69.8 (3′-C), 55.8 (17′-C), 55.4 (14′-C), 54.4 (7′-C), 53.8 (9′-C), 44.5 (13′-C), 44.1 (12′-C), 43.8 (24′-C), 39.8 (10′-C), 39.2 (8′-C), 38.5 (4′-C), 36.0 (22′-C), 35.5 (20′-C), 32.4 (1′-C), 28.2 (2′-C), 27.7 (16′-C), 25.0 (25′-C), 23.5 (15′-C), 23.3 (CH_3_CO), 21.8 (26′-C), 21.6 (23′-C), 21.5 (27′-C), 19.8 (11′-C), 19.0 (5-CH_3_ and 6-CH_3_), 17.9 (21′-C), 17.3 (19′-C), 11.5 (18′-C); HREIMS *m/z*: 577.4390 [M + H]^+^ (calcd. for C_37_H_57_N_2_O_3_, 577.4369).

2-(3β′,5β′-Dihydroxy-6′-B-norcholesteryl)-5-methoxybenzimidazole (**11a**) Brownish oil, yield: 49%. IR (KBr) ν/cm^−1^: 3420, 2942, 1629, 1516, 1452, 1377, 1198, 1152, 1028; ^1^H NMR (CDCl_3_, 300 MHz) δ: 0.65 (3H, s, 18′-CH_3_), 0.86 (3H, d, *J* = 6.6, 26′- or 27′-CH_3_), 0.87 (3H, d, *J* = 6.6, 26′- or 27′-CH_3_), 0.92 (3H, d, *J* = 6.6, 21′-CH_3_), 1.10 (3H, s, 19′-CH_3_), 2.16–2.05 (2H, m, C4′-H and C8′-H), 2.71 (1H, d, *J* = 10.8, C7′-H), 3.83 (3H, s, 5-OCH_3_), 3.98–3.91 (1H, m, C3′-H), 6.85 (1H, dd, *J* = 8.7, 2.4, C6-H), 7.06 (1H, s, C4-H), 7.44 (1H, d, *J* = 8.7, C7-H); ^13^C NMR (CDCl_3_, 75 MHz) δ: 156.1 (2-C), 155.3 (5-C), 155.2 (8-C), 130.1 (9-C), 120.4 (7-C), 111.53 (6-C), 111.52 (4-C), 82.2 (5′-C), 67.0 (3′-C), 56.2 (17′-C), 55.8 (-OCH_3_), 55.5 (14′-C), 54.7 (7′-C), 54.2 (9′-C), 45.6 (13′-C), 45.2 (12′-C), 44.6 (24′-C), 42.6 (10′-C), 39.7 (4′-C), 39.4 (8′-C), 36.2 (22′-C), 35.5 (20′-C), 31.2 (1′-C), 29.7 (2′-C), 28.5 (16′-C), 28.0 (25′-C), 24.2 (15′-C), 23.8 (23′-C), 22.8 (27′-C), 22.5 (26′-C), 21.7 (11′-C), 18.8 (21′-C), 17.8 (19′-C), 12.5 (18′-C); HREIMS *m/z*: 537.4057 [M + H]^+^ (calcd. for C_34_H_53_N_2_O_3_, 537.4056).

2-(3β′-Acetoxy-5β′-hydroxy-6′-B-norcholesteryl)-5-methoxybenzimidazole (**11b**) Yellow solid, yield: 29%, m.p.: 135–137 °C. IR (KBr) ν/cm^−1^: 3456, 2942, 2863, 1716, 1629, 1594, 1486, 1417, 1372, 798; ^1^H NMR (CDCl_3_, 300 MHz) δ: 0.65 (3H, s, 18′-CH_3_), 0.85 (6H, d, *J* = 6.6, 26′- or and 27′-CH_3_), 0.91 (3H, d, *J* = 6.3, 21′-CH_3_), 1.09 (3H, s, 19′-CH_3_), 1.96 (3H, s, 3-COCH_3_), 2. 20 (1H, q, *J* = 6.3, C8′-H), 2.76 (1H, d, *J* = 10.8, C7′-H), 3.68 (1H, br s, -OH), 3.83 (3H, s, 5-OCH_3_), 5.01-4.93 (1H, m, C3′-H), 6.85 (1H, dd, *J* = 8.7, 2.1, C6-H), 7.19 (1H, br s, C4-H), 7.55 (1H, br s, C7-H); ^13^C NMR (CDCl_3_, 75 MHz) δ: 170.7 (3′-C=O), 156.1 (2-C), 155.3 (5-C), 155.2 (8-C), 130.1 (9-C), 120.4 (7-C), 111.53 (6-C), 111.52 (4-C), 81.1 (5′-C), 70.0 (3′-C), 55.8 (17′-C), 55.7 (-OCH_3_), 55.4 (14′-C), 54.7 (7′-C), 54.4 (9′-C), 45.1 (13′-C), 44.8 (12′-C), 44.5 (24′-C), 39.7 (10′-C), 39.4 (4′-C), 39.3 (8′-C), 36.2 (22′-C), 35.5 (20′-C), 32.8 (1′-C), 29.7 (2′-C), 28.5 (16′-C), 28.0 (25′-C), 25.4 (15′-C), 23.8 (23′-C), 22.8 (27′-C), 22.5 (26′-C), 21.9 (CH_3_CO), 21.4 (11′-C), 18.8 (21′-C), 18.1 (19′-C), 12.5 (18′-C); HREIMS *m/z*: 579.4162 [M + H]^+^ (calcd. for C_36_H_55_N_2_O_4_, 579.4142).

#### 3.1.2. Compounds **12a**–**12b** Were Prepared Similarly According to the Procedure of **7**–**11**, but Using 4-Trifluoromethyl-*O*-phenylenediamine as Reagent

*N*-(2′-amino-5′-trifluoromethyl)phenyl-3β-hydroxy-B-norcholestano[7,5-*d*] isoxazolidine (**12a**) Brownish oil, yield: 40%; IR (KBr) ν/cm^−1^: 3424, 2947, 1627, 1524, 1447, 1329, 1232, 1110, 931; ^1^H NMR (CDCl_3_, 300 MHz) δ: 0.66 (3H, s, 18-CH_3_), 0.87 (6H, d, *J* = 6.6, 26- and 27-CH_3_), 0.94 (3H, d, *J* = 6.3, 21-CH_3_), 1.08 (3H, s, 19-CH_3_), 2.08 (1H, d, *J* = 12.9, C6-H), 2.17 (1H, q, *J* = 10.8, C6-H), 2.79 (1H, d, *J* = 10.5, C7-H), 3.88 (1H, br s, C3-H), 4.30 (1H, br s, -OH), 7.44 (1H, d, *J* = 8.4, 6-ArH), 7.60 (1H, br s, 3-ArH), 7.88 (1H, br s, 5-ArH); ^13^C NMR (CDCl_3_, 75 MHz) δ: 158.3 (1-PhC), 130.3 (CF_3_), 126.7 (2-PhC), 124.7 (4-PhC), 124.2 (5-PhC), 123.1 (3-PhC), 119.1 (6-PhC), 82.5 (5-C), 70.0 (3-C), 56.1 (17-C), 55.3 (14-C), 55.1 (9-C), 54.7 (7-C) 45.8 (8-C), 45.3 (13-C), 44.6 (10-C), 42.3 (6-C), 39.5 (12-C), 39.4 (24-C), 36.2 (4-C), 35.5 (20-C), 31.6 (22-C), 29.7 (1-C), 28.4 (2-C), 28.3 (16-C), 28.0 (25-C), 24.2 (15-C), 23.7 (23-C), 22.8 (26-C), 22.5 (27-C), 21.7 (11-C), 18.8 (21-C), 17.5 (19-C), 12.4 (18-C); HREIMS *m/z*: 577.3902 [M + H]^+^ (calcd. for C_34_H_50_F_3_N_2_O_2_, 577.3981).

*N*-(2′-amino-5′-trifluoromethyl)phenyl-3β-acetoxy-B-norcholestano[7,5-*d*] isoxazolidine (**12b**) Yellow solid, yield: 87.8%, m.p.: 155–157 °C. IR (KBr) ν/cm^−1^: 3461, 2948, 2858, 1711, 1629, 1527, 1417, 1362, 1330, 1263, 1115, 1052; ^1^H NMR (CDCl_3_, 300 MHz) δ: 0.61 (3H, s, 18-CH_3_), 0.85 (6H, d, *J* = 6.6, 26- and 27-CH_3_), 0.91 (3H, d, *J* = 6.3, 21-CH_3_), 1.10 (3H, s, 19-CH_3_), 1.86 (3H, s, COCH_3_), 2.28 (2H, q, *J* = 10.8, C6-H), 2.96 (1H, d, *J* = 11.7, C7-H), 3.38 (1H, br s, -NH), 5.02–4.89 (1H, m, C3-H), 7.45 (1H, d, *J* = 8.4, 6-ArH), 7.74 (1H, s, 3-ArH), 7.98 (1H, br s, 5-ArH); ^13^C NMR (CDCl_3_, 75 MHz) δ: 170.6 (COCH_3_), 157.3 (1-PhC), 130.3 (CF_3_), 126.7 (2-PhC), 124.6 (4-PhC), 124.2 (5-PhC), 123.1 (3-PhC), 119.1 (6-PhC), 81.6 (5-C), 69.7 (3-C), 55.7 (17-C), 55.4 (14-C), 54.7 (9-C), 54.6 (7-C), 44.9 (8-C), 44.8 (10-C), 44.5 (13-C), 39.7 (6-C), 39.4 (12-C), 39.0 (24-C), 36.1 (22-C), 35.5 (20-C), 32.7 (4-C), 29.7 (1-C), 28.4 (16-C), 28.0 (25-C), 25.4 (2-C), 24.0 (15-C), 23.7 (23-C), 22.8 (26-C), 22.5 (27-C), 21.9 (11-C), 21.1 (CH_3_CO), 18.8 (21-C), 18.2 (19-C), 12.4 (18-C); HREIMS *m/z*: [M + H]^+^ 619.3996 (calcd. for C_36_H_54_F_3_N_2_O_3_, 619.4087).

#### 3.1.3. Compound **13** Was Prepared Similarly According to the Procedure of **7**–**11**, but Using 2-Mercaptophenylamine as Reagent

2-(3β′,5β′-Dihydroxy-6′-B-norcholesteryl)benzothiazole (**13**) Light yellow solid, yield: 55%, m.p.: 180–182 °C. IR (KBr) ν/cm^−1^: 3453, 2920, 2726, 1674, 1589, 1462, 1377, 1165, 1073, 951; ^1^H NMR (CDCl_3_, 300 MHz) δ: 0.69 (3H, s, 18′-CH_3_), 0.87 (3H, d, *J* = 6.6, 26′- or 27′-CH_3_), 0.87 (3H, d, *J* = 6.6, 26′- or 27′-CH_3_), 0.94 (3H, d, *J* = 6.3, 21′-CH_3_), 1.13 (3H, s, 19′-CH_3_), 2.14-2.06 (2H, m, C4′-H), 2.25 (1H, q, *J* = 10.8, C8′-H), 3.04 (1H, d, *J* = 9.6, C7′-H), 3.30 (1H, br s, -OH), 3.95 (1H, s, -OH), 4.10–4.04 (1H, m, C3′-H), 7.37 (1H, td, *J* = 8.1, 1.5, 5-ArH), 7.47 (1H, td, *J* = 7.8, 1.5, 6-ArH), 7.85 (1H, d, *J* = 7.5, 4-ArH), 7.99 (1H, d, *J* = 7.8, 7-ArH); ^13^C NMR (CDCl_3_, 75MHz) δ: 173.1 (2-C), 153.4 (8-C), 134.5 (9-C), 126.0 (5-C), 124.7 (6-C), 122.7 (7-C), 121.4 (4-C), 83.2 (5′-C), 67.1 (3′-C), 58.3 (17′-C), 56.8 (14′-C), 55.5 (7′-C), 51.8 (9′-C), 47.2 (13′-C), 45.3 (8′-C), 44.8 (12′-C), 44.1 (24′-C), 39.7 (10′-CH_2_), 39.5 (4′-C), 36.2 (22′-C), 35.6 (20′-C), 28.9 (1′-C), 28.5 (2′-C), 28.4 (16′-C), 28.0 (25′-C), 24.7 (15′-C), 23.8 (23′-C), 22.8 (27′-C), 22.5 (26′-C), 21.6 (11′-C), 18.8 (21′-C), 18.2 (19′-C), 12.5 (18′-C); HREIMS *m/z*: 524.3562 [M + H]^+^ (calcd. for C_33_H_50_NO_2_S, 524.3562).

#### 3.1.4. 3β,5β-Dihydroxy-6-(*N*-3′-(2-amino)pyridyl)imine-B-norcholestane (**15**)

Compound **15** was prepared similarly according to the procedure of **7**–**11**, but using 2,3-diaminopyridine as reagent.

Brown oil, Yield: 49%. IR (KBr) ν/cm^−1^: 3454, 2920, 1716, 1671, 1589, 1462, 1377, 1165, 1073, 1013; ^1^H NMR (CDCl_3_, 300 MHz) δ: 0.67 (3H, s, 18-CH_3_), 0.84 (6H, d, *J* = 6.9, 26- and 27-CH_3_), 0.90 (3H, d, *J* = 6.3, 21-CH_3_), 0.95 (3H, s, 19-CH_3_), 2.35–2.30 (1H, m, C7-H), 3.38 (1H, s, -OH), 4.12–4.07 (1H, m, C_3_-H), 4.34 (2H, br s, -NH_2_), 5.00 (1H, s, -OH), 6.52 (1H, dd, *J* = 7.5, 5.1, 5′-PhH), 6.94 (1H, dd, *J* = 7.5, 1.5, 4′-PhH), 7.77 (1H, dd, *J* = 5.1, 1.2, 6′-PhH), 7.85 (1H, d, *J* = 6.6, C6-H); ^13^C NMR (CDCl_3_, 75 MHz) δ: 170.7 (6-C), 153.2 (2-PhC), 144.4 (6-PhC), 133.5 (3-PhC), 125.0 (4-PhC), 113.8 (5-PhC), 84.3 (5-C), 66.7 (3-C), 60.4 (17-C), 59.4 (14-C), 56.2 (7-C) 55.6 (9-C), 51.4 (13-C), 45.5 (10-C), 44.7 (8-C), 44.1 (12-C), 42.6 (24-C), 39.7 (4-C), 39.4 (22-C), 36.2 (20-C), 35.6 (1-C), 28.5 (2-C), 28.0 (16-C), 24.9 (25-C), 23.8 (15-C), 22.8 (26-C), 22.6 (27-C), 21.7 (23-C), 21.0 (11-C), 18.8 (21-C), 14.2 (19-C), 12.5 (18-C); HREIMS *m/z*: 510.4090 [M + H]^+^ (calcd. for C_32_H_52_N_3_O_2_, 510.4060).

### 3.2. Biological Assays

#### 3.2.1. Materials

Stock solutions of the compounds were prepared in sterile dimethyl sulfoxide (DMSO) (Sigma, St. Louis, MO, USA) at a concentration of 10 mg/mL and afterward diluted with complete nutrient medium (RPMI-1640) supplemented with 10% heat inactivated fetal bovine serum and 0.1 g/L penicillin G + 0.1 g/L streptomycin sulfate.

#### 3.2.2. Cell Culture

HeLa, A549, HEPG2 cancer cells and HEK293T cells were grown in the medium (RPMI-1640) supplemented with 10% heat inactivated fetal bovine serum and 0.1 g/L penicillin G + 0.1 g/L streptomycin sulfate in a humidified atmosphere of 5% CO_2_ at 37 °C.

#### 3.2.3. Assay for Cell Viability

The anticancer activity *in vitro* was measured using the MTT assay. Briefly, cells (1~2 × 10^4^ cells per well) were seeded in 96-wells plates for 24 h. Different concentrations of the test compound were added to the cells. An equal amount of DMSO was added to the cells used as negative controls. Triplicate wells were prepared for each individual dose. After reincubated for 72 h, the cells were washed with sterile phosphate buffer saline (PBS). 190 µL of RPMI-1640 and 10 µL of the tetrazolium dye (MTT) (5 mg/mL) solution were added to each well, and the cells were incubated for additional 4 h. After the supernatant was discarded, 200 µL of DMSO was added to dissolve the purple formazan crystals formed. The absorbance values (*A*) at 492 nm were determined using a MLLTISKAN MK3 analysis spectrometer (Thermo Scientific, Shanghai, China). The IC_50_ values were calculated as the concentration of drug yielding 50% cell survival.

#### 3.2.4. Annexin V Staining Assay

Apoptosis was detected with an annexin V-FITC kit purchased from BD Pharmingen (San Diego, CA, USA) according to the manufacturer’s instructions. HeLa cells were seeded in 35 mm culture dishes and allowed to attach overnight. The cells were treated with **9b**, **10a** and **11a** for 24 h, respectively, collected, and washed twice with PBS. To detect early and late apoptosis, both adherent and floating cells were harvested together and resuspended in annexin V binding buffer at a concentration of 10^6^ cells/mL. Subsequently, 5 μL of FITC-conjugated annexin V and 5 μL of propidium iodide were added to 100 μL of the cell suspension (10^5^ cells). The cells were incubated for 15 min at room temperature in the dark. Finally, 400 μL of annexin V binding buffer was added to each tube, and cells were analyzed by a two-color cytometry using FACS Calibur flow cytometry (Becton Dickinson, Biosciences, Franklin Lakes, NJ, USA).

## 4. Conclusions

We synthesized some novel B-norcholesteryl benzimidazole and benzothiazole derivatives. The antiproliferative activity of the compounds against human cervical carcinoma (HeLa), human lung carcinoma (A549), human liver carcinoma cells (HEPG2) and normal kidney epithelial cells (HEK293T) was assayed. The results showed that some B-norcholesteryl benzimidazole compounds exhibited an excellent antiproliferative activity and almost inactive to normal kidney epithelial cells (HEK293T). In addition, the results revealed that the benzimidazole group was a better substituent than benzothiazole group for increasing the antiproliferative activity of compounds. The most potent compound **9b** with the structure of 6-benzimidazole exhibited excellent antiproliferative activities with an IC_50_ value of 4.7, 11.9 and 4.2 μM against HeLa, A549 and HEPG2 cells, respectively, and was able to effectively induce tumor cells apoptotic. The results suggest that B-norcholesteryl derivatives based on benzimidazole group may constitute a novel class of antiproliferative agents, which deserve further study.

## Supplementary Files

Supplementary File 1
