# Supplementary material for: Synthesis and in Vitro Antiproliferative Evaluation of Some B-norcholesteryl Benzimidazole and Benzothiazole Derivatives
Source: Mar Drugs. 2015 Apr 22;13(4):2488–504. doi: 10.3390/md13042488 (PMC4413222; doi:10.3390/md13042488)
Supplement: Supplementary File 1 [file marinedrugs-13-02488-s001.pdf]

Supplementary Information

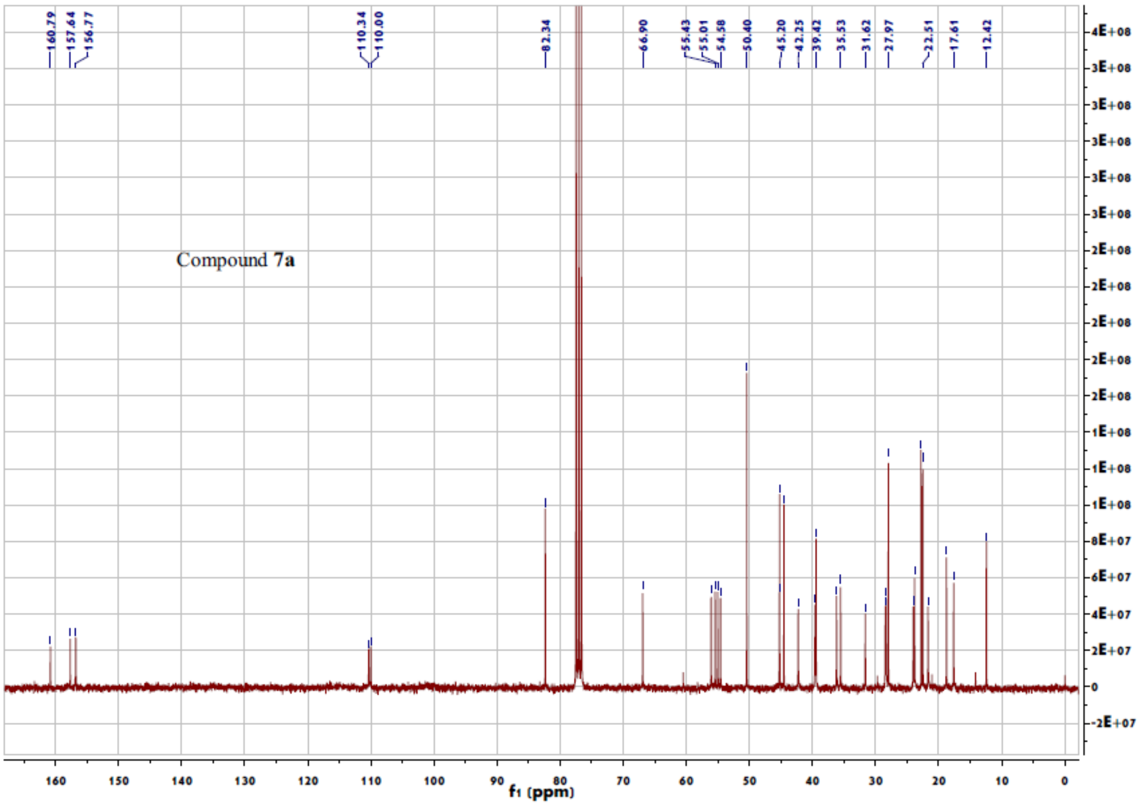

Figure S1. Compound **7a** ^{13}C NMR.

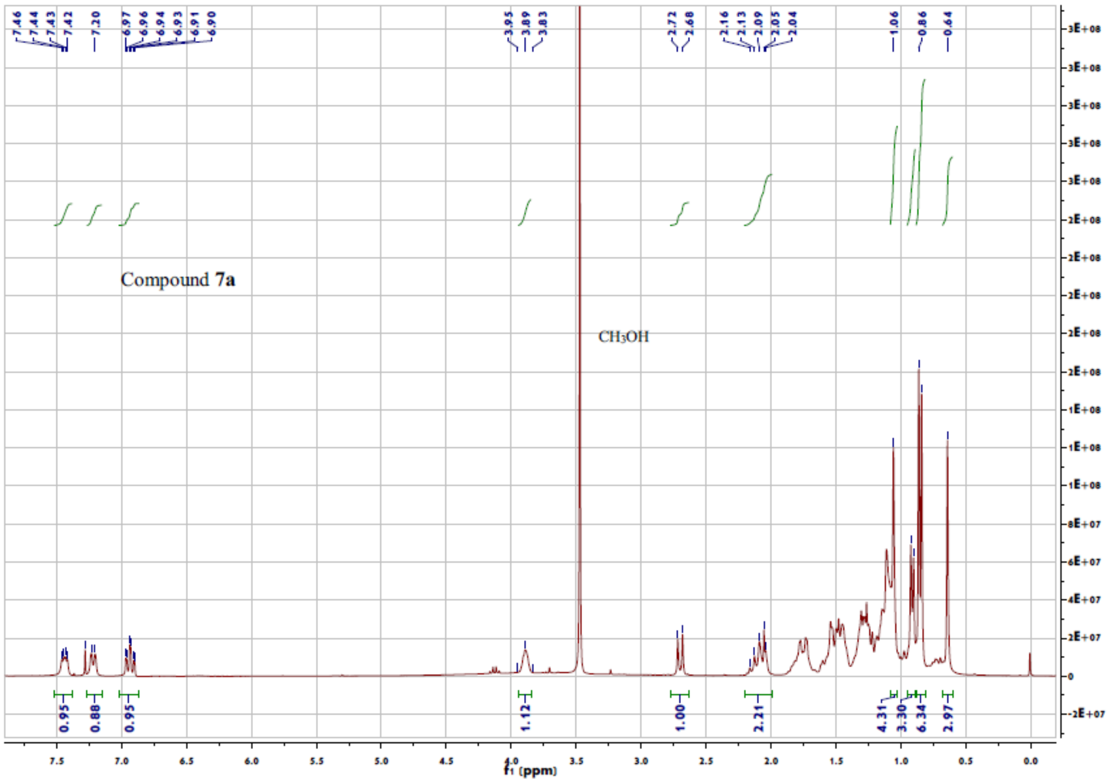

Figure S2. Compound **7a** ^{1}H NMR.

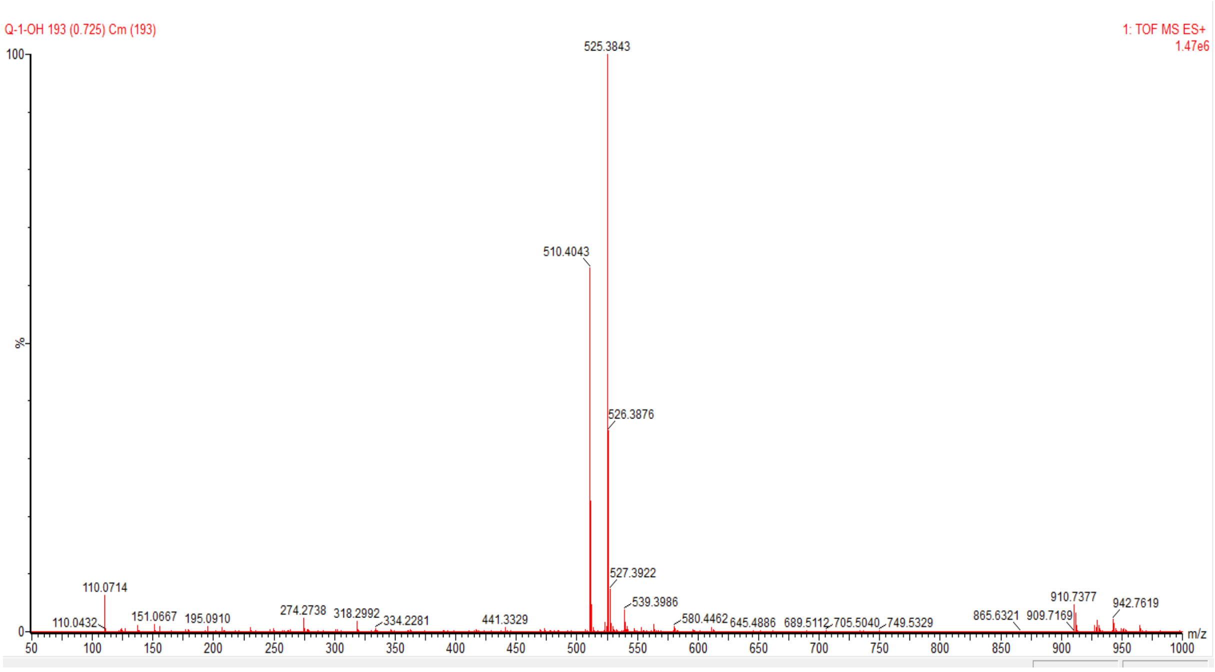

Figure S3. Compound **7a** HREIMS.

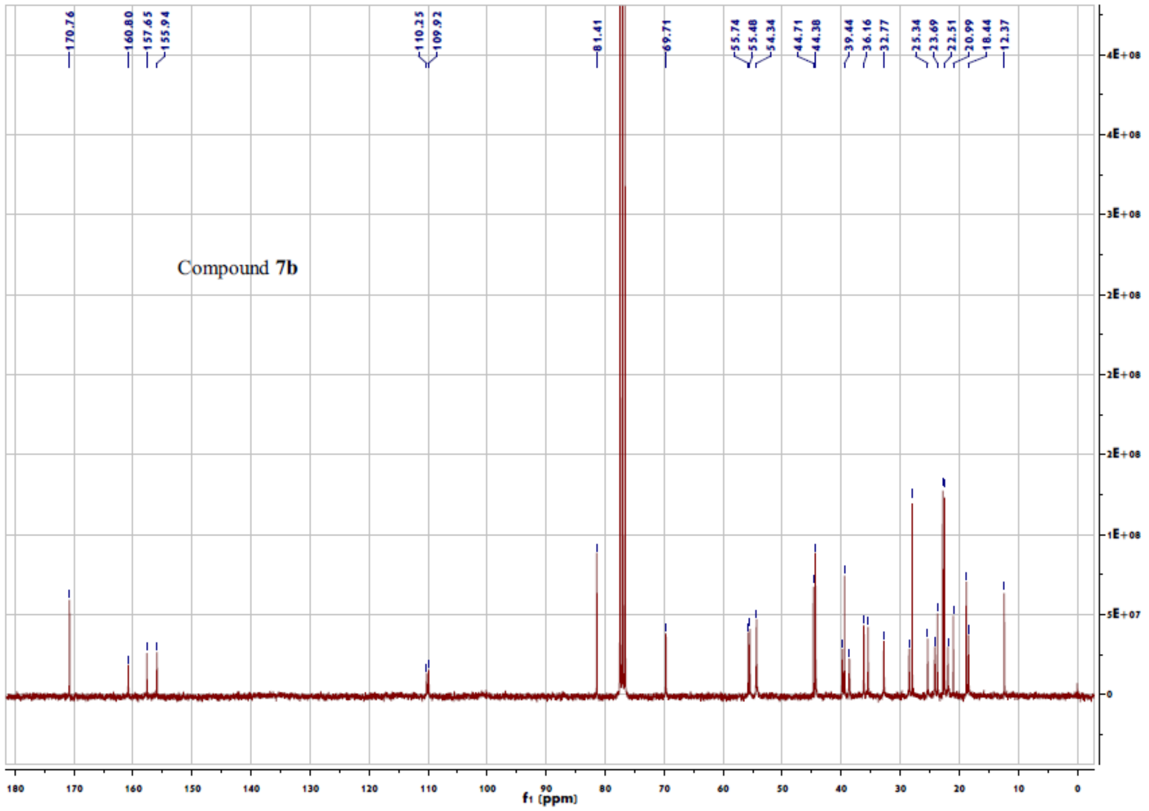

Figure S4. Compound **7b** ^{13}C NMR.

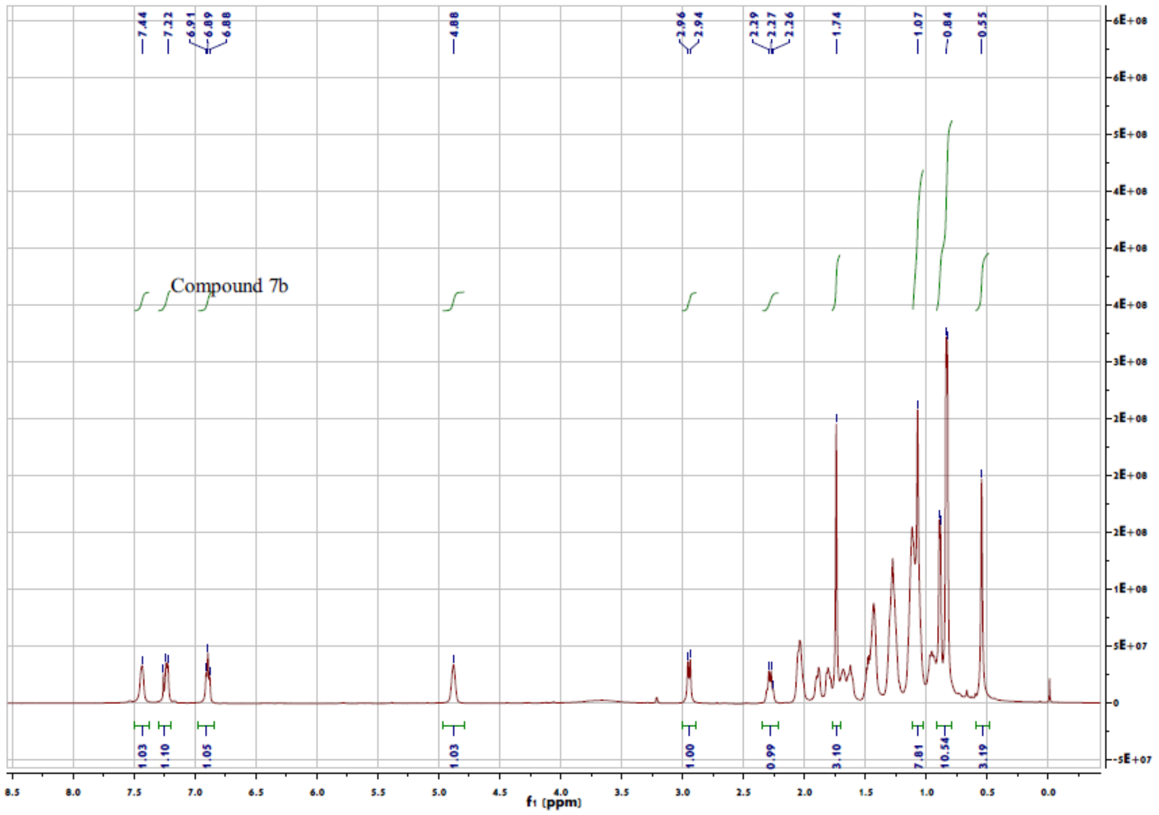

Figure S5. Compound **7b** ^{1}H NMR.

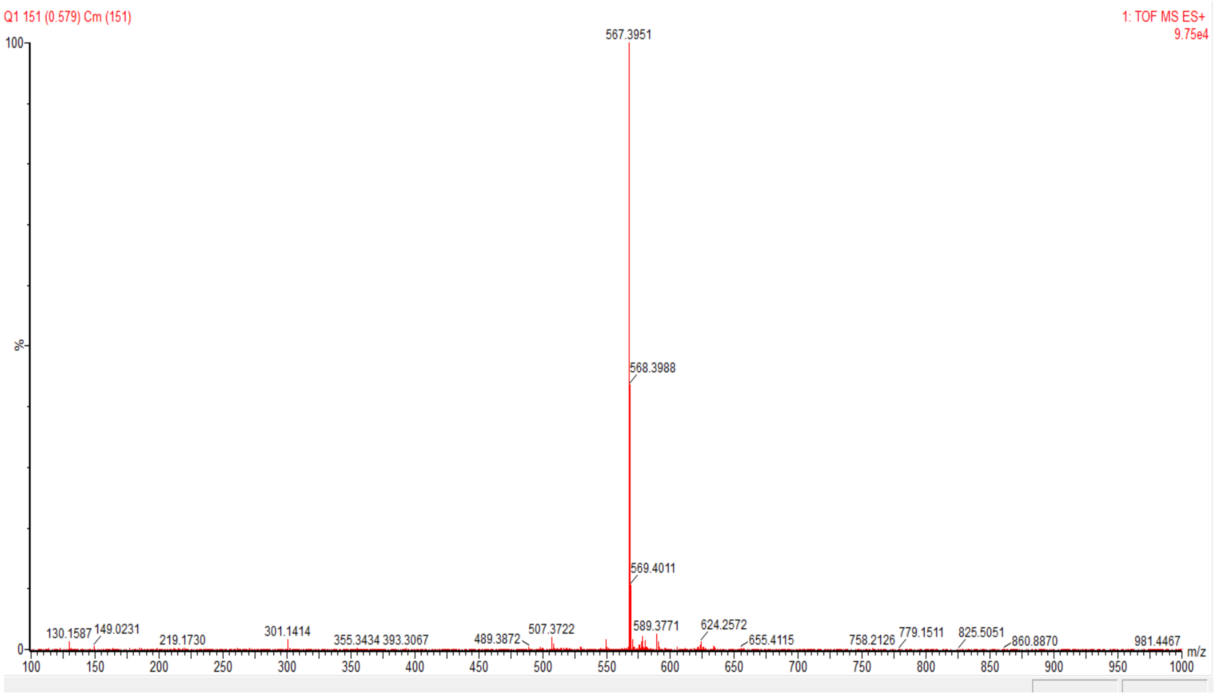

Figure S6. Compound **7b** HREIMS.

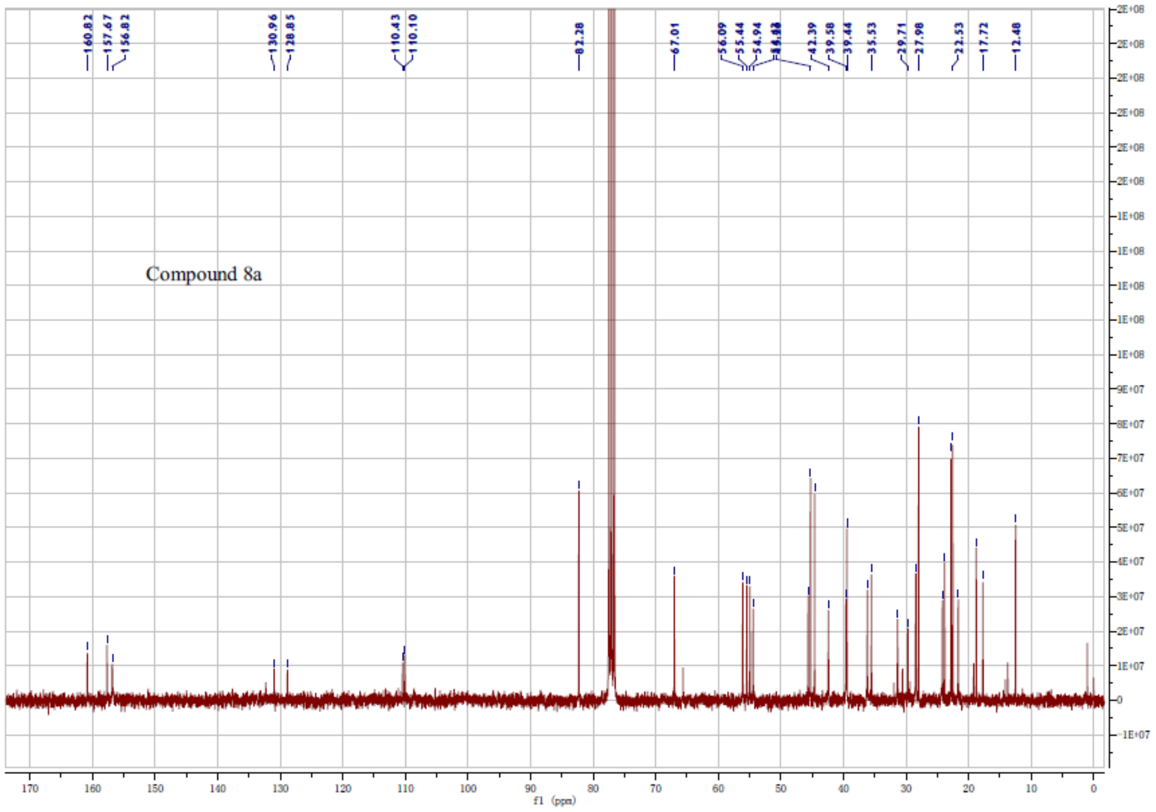

Figure S7. Compound **8a** ^{13}C NMR.

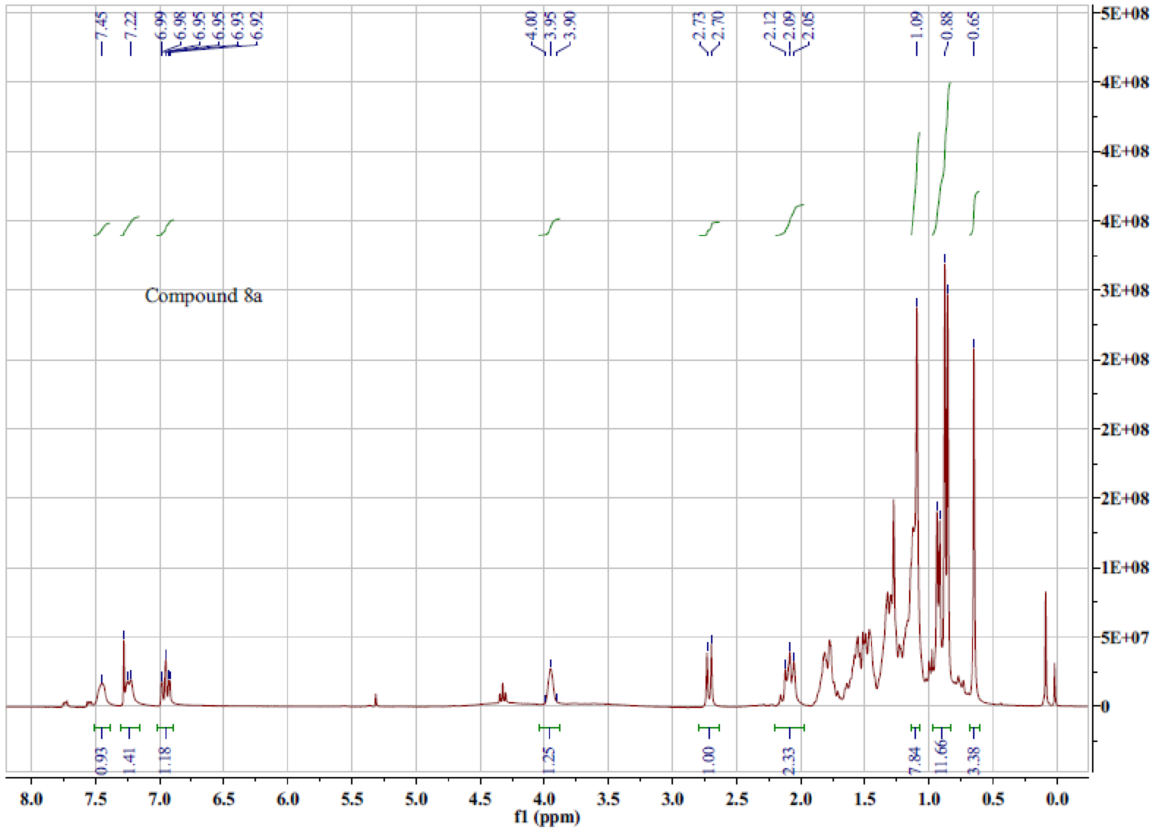

Figure S8. Compound **8a** ^{1}H NMR.

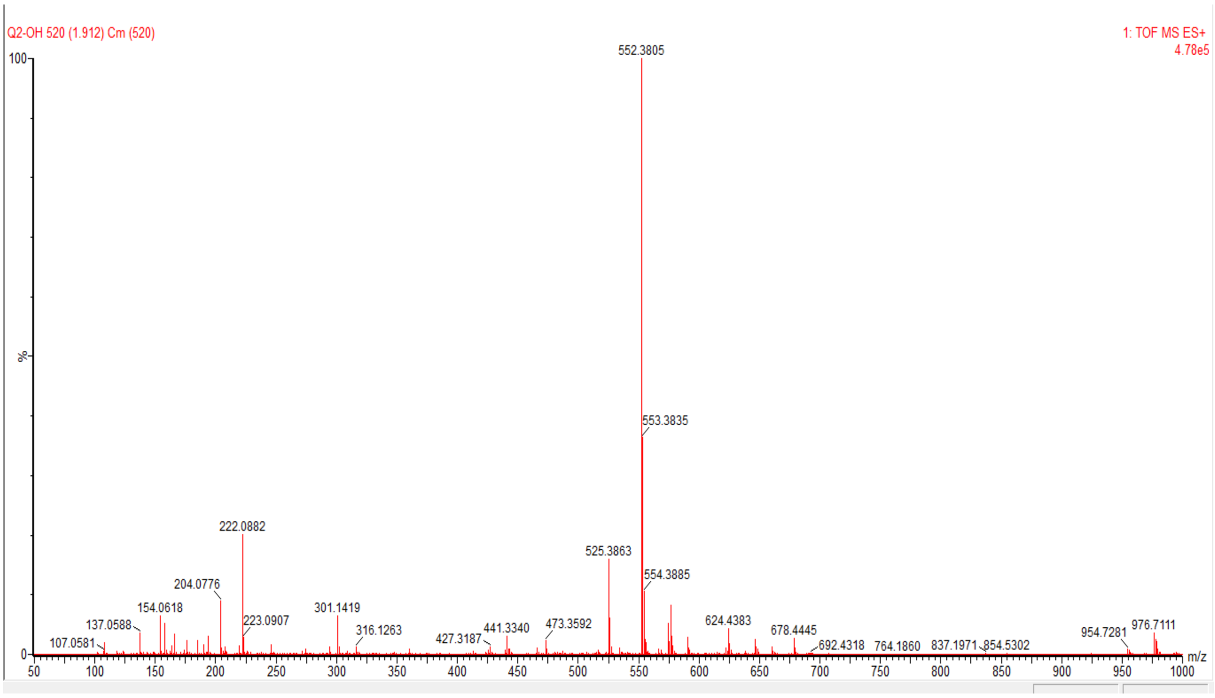

Figure S9. Compound **8a** REIMS.

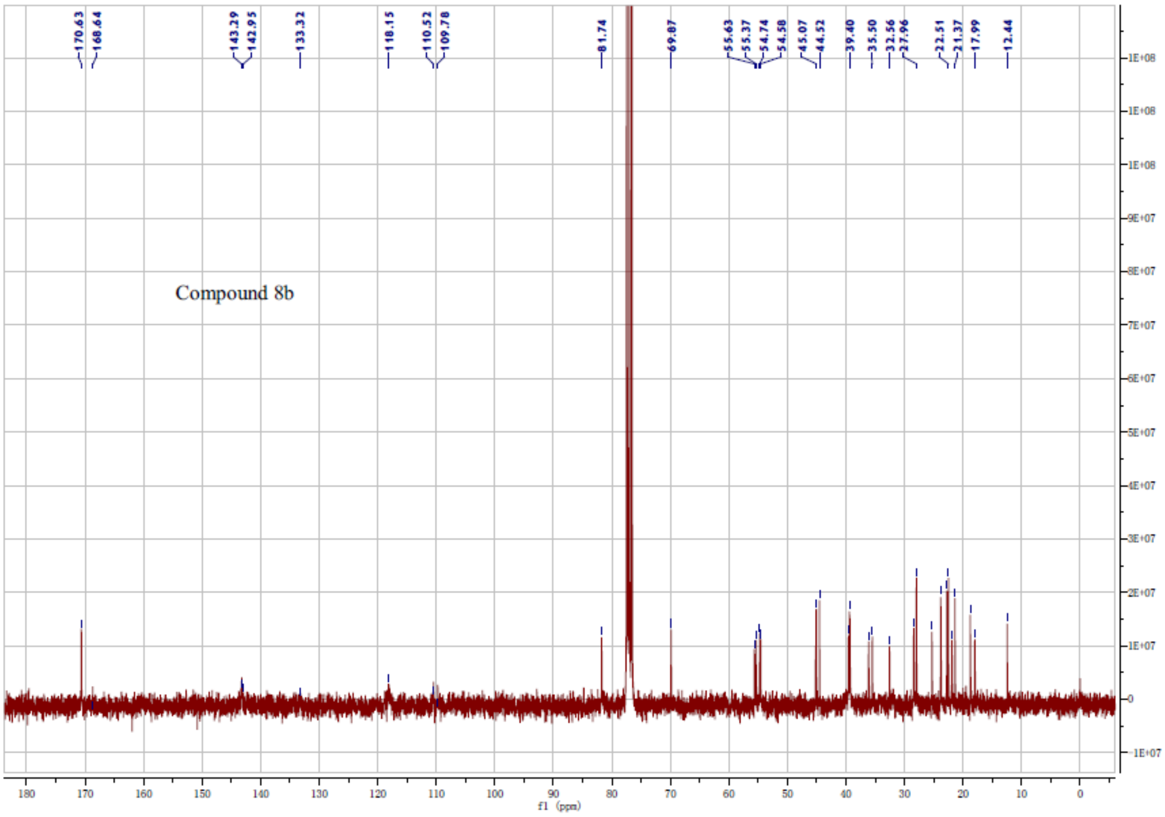

Figure S10. Compound **8b** ^{13}C NMR.

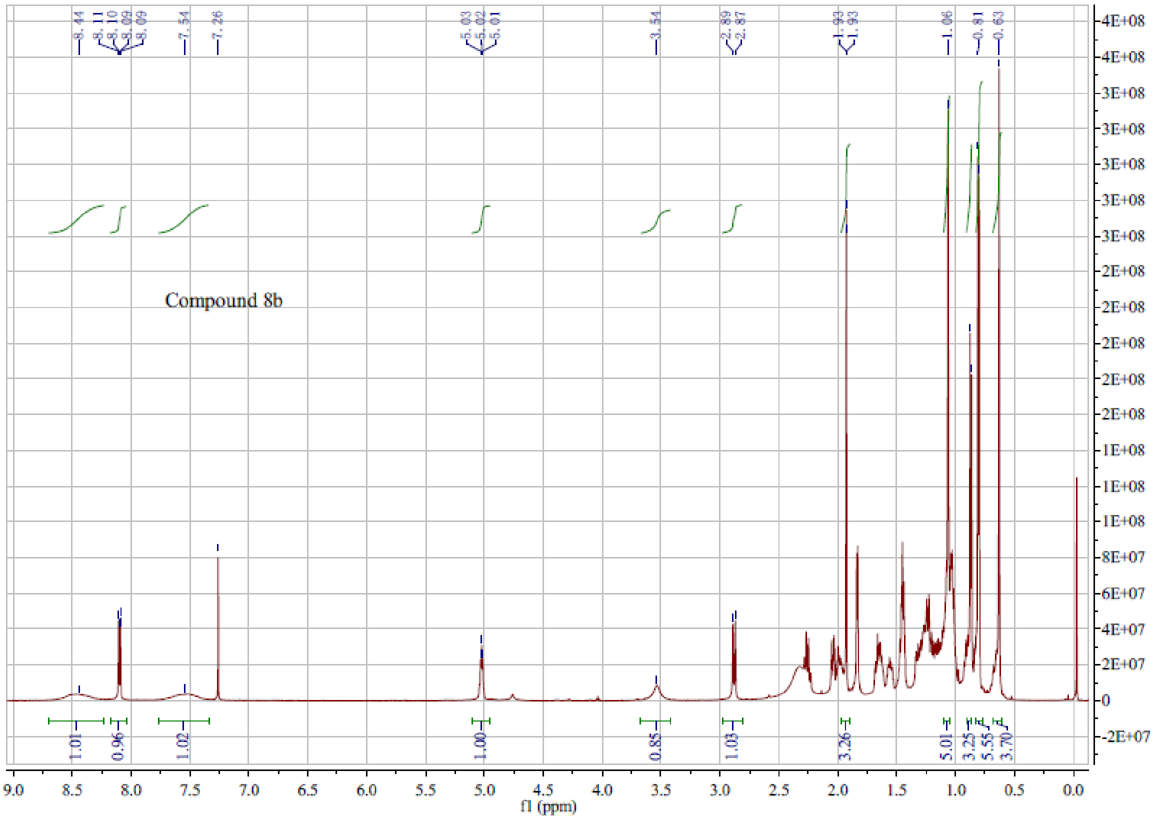

Figure S11. Compound **8b** ^{1}H NMR.

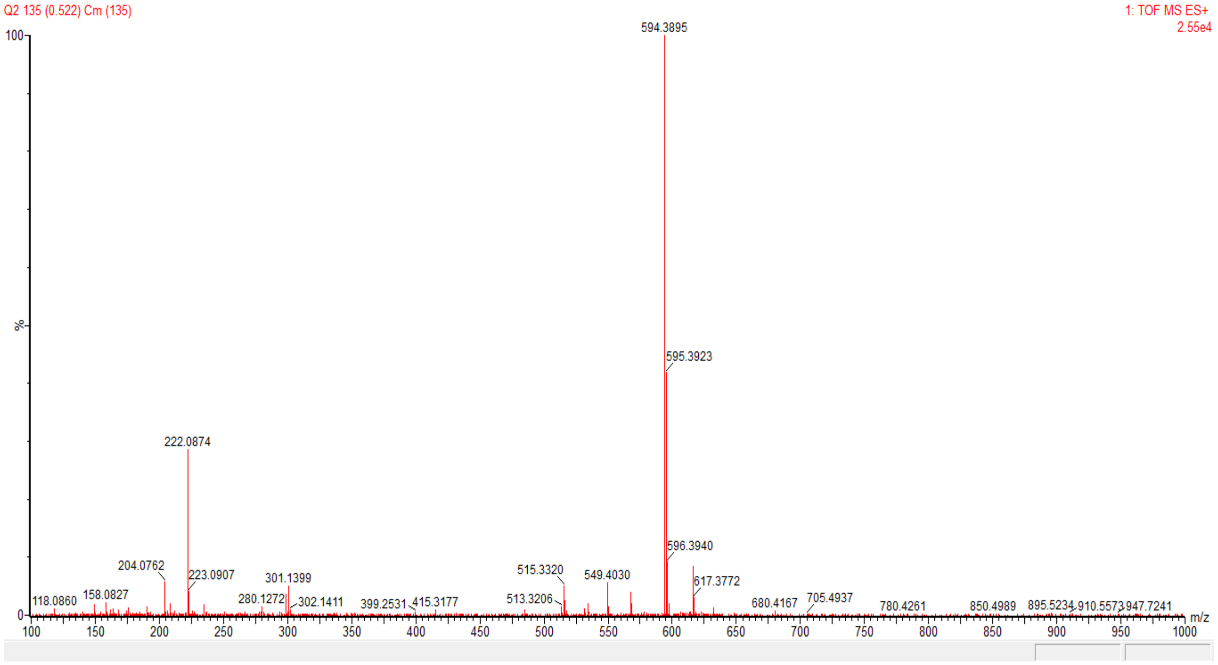

Figure S12. Compound **8b** HREIMS.

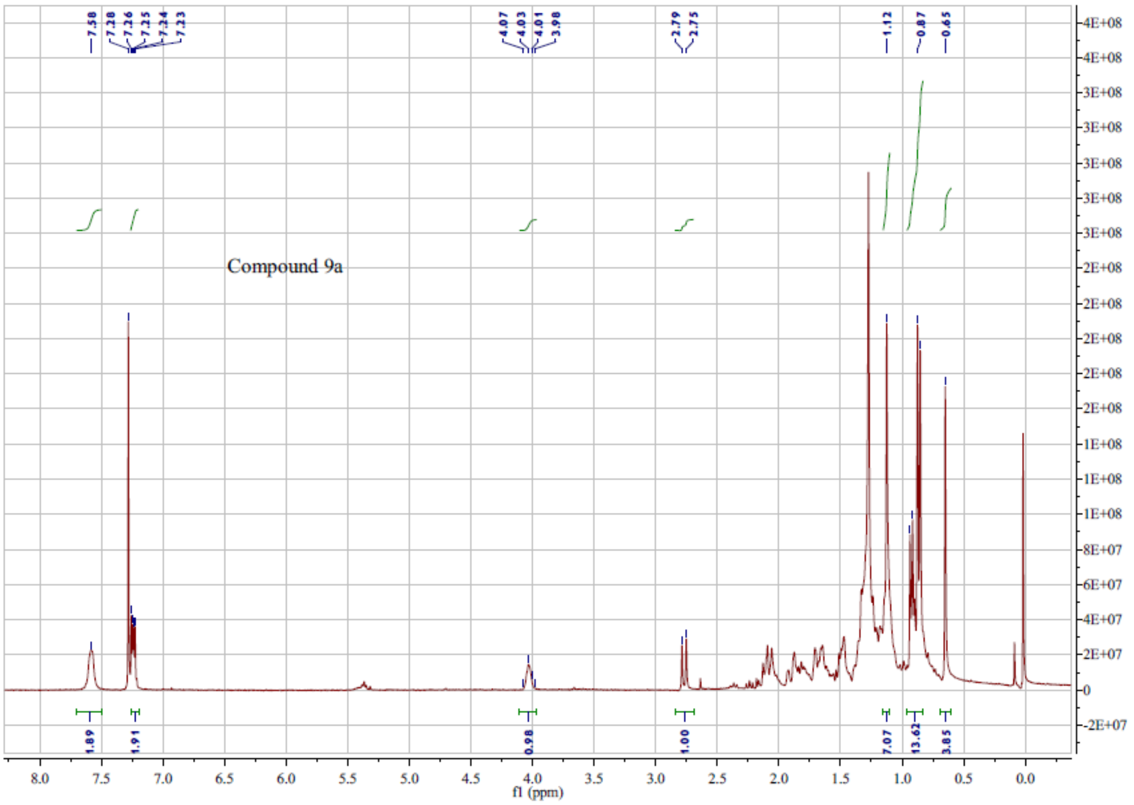

Figure S13. Compound **9a** ^{1}H NMR.

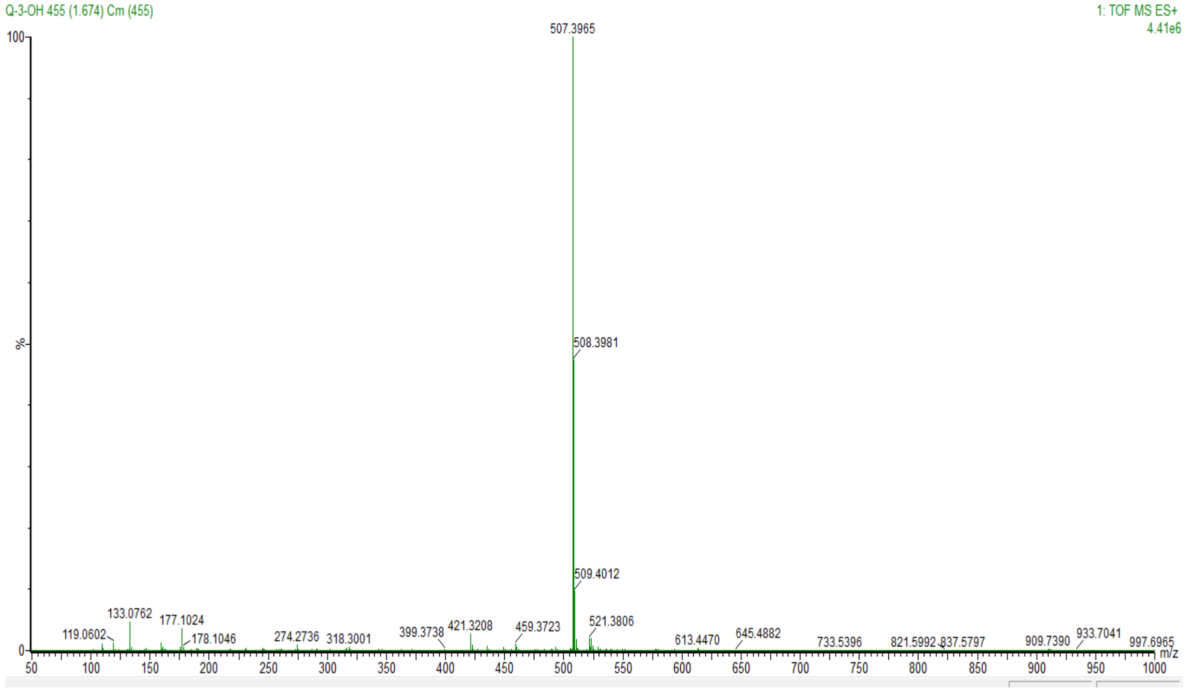

Figure S14. Compound **9a** HREIMS.

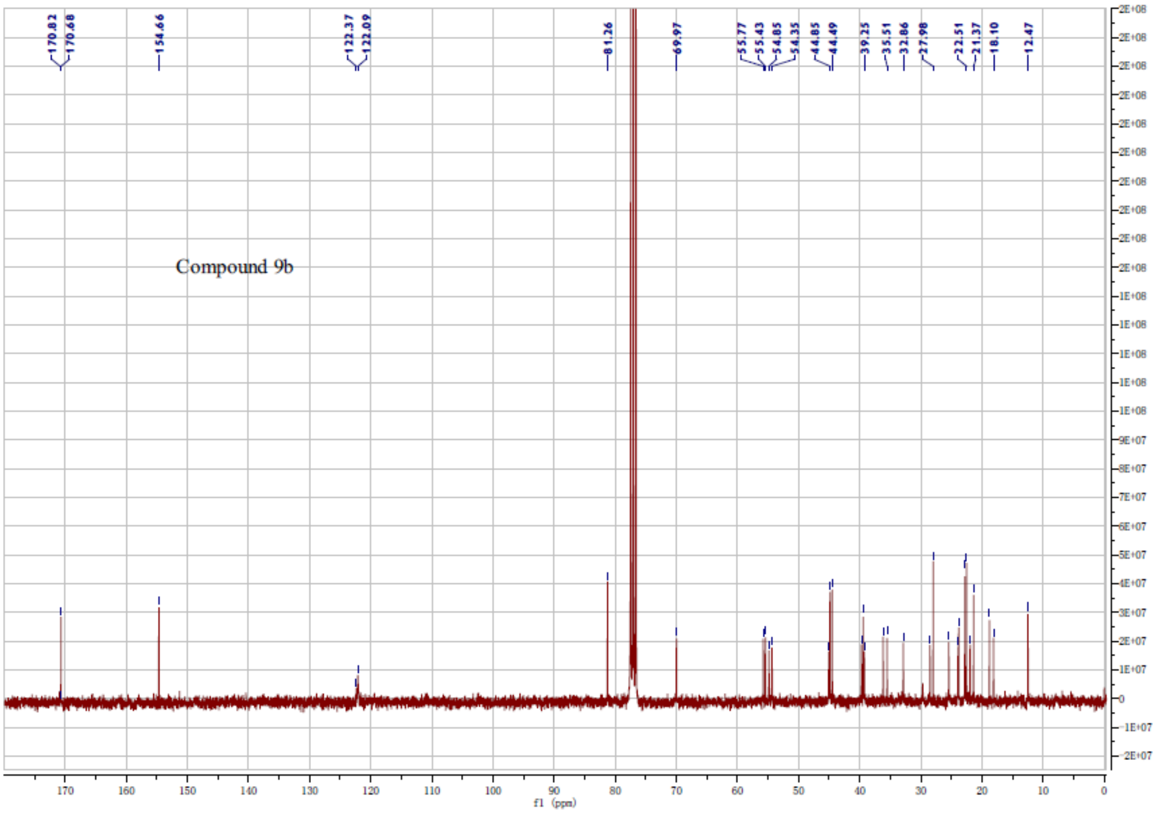

Figure S15. Compound **9b** ^{13}C NMR.

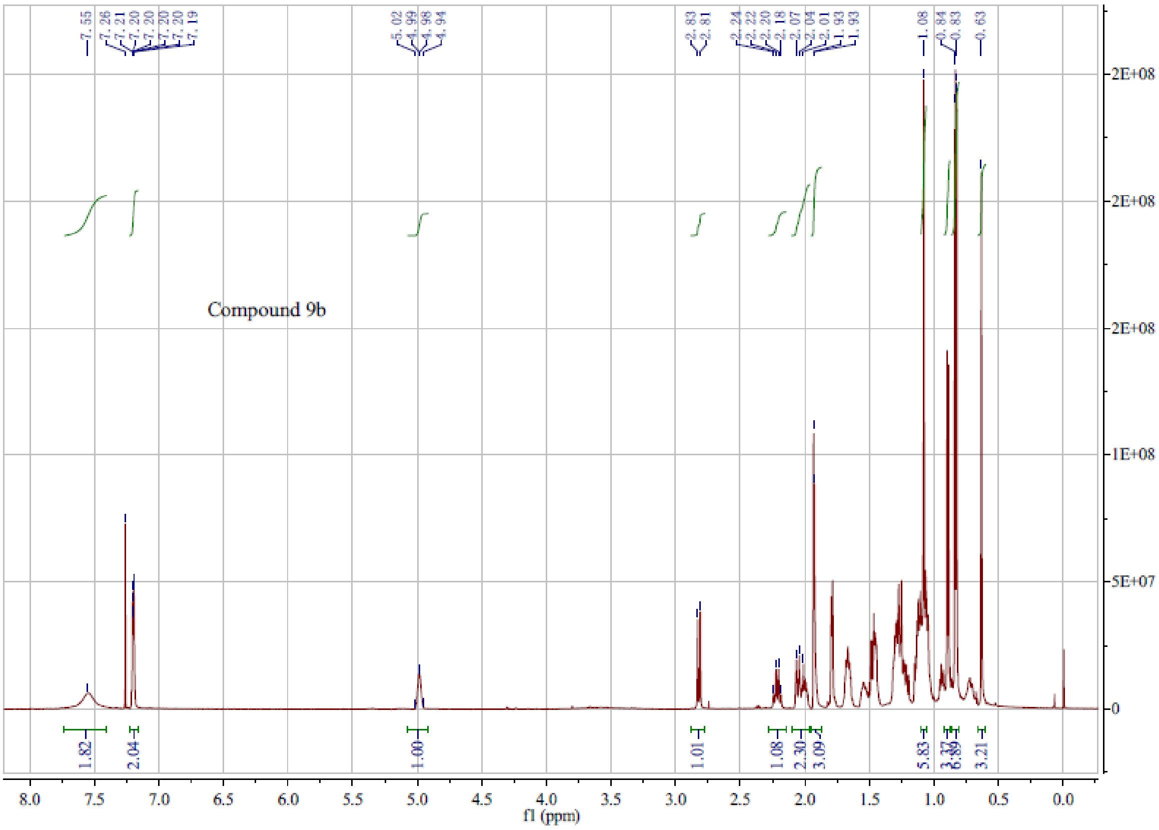

Figure S16. Compound **9b** ^{1}H NMR.

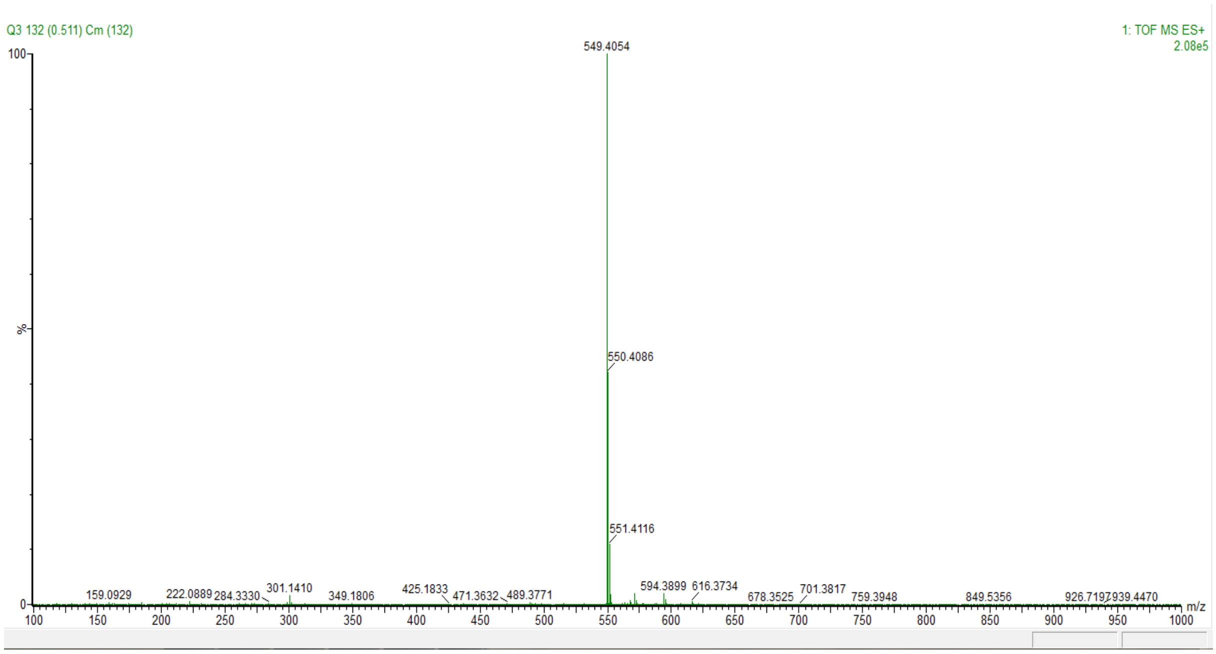

Figure S17. Compound **9b** HREIMS.

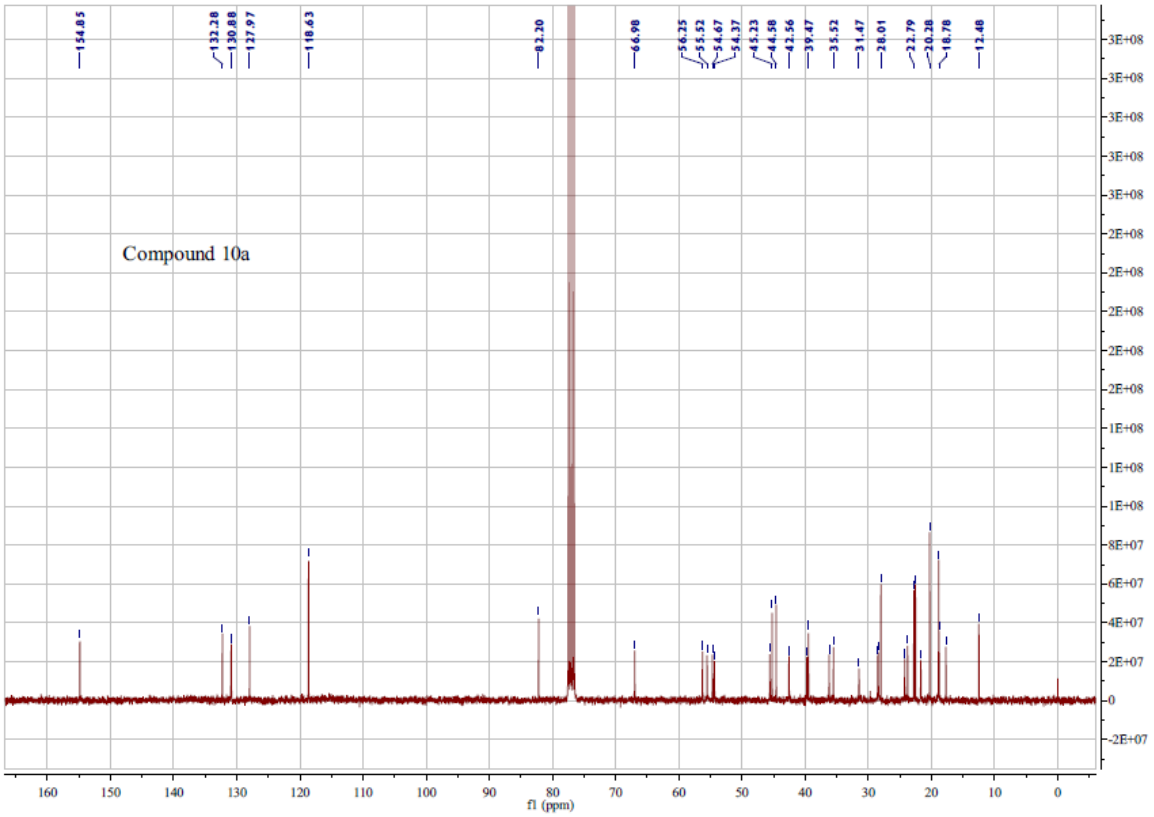

Figure S18. Compound **10a** ^{13}C NMR.

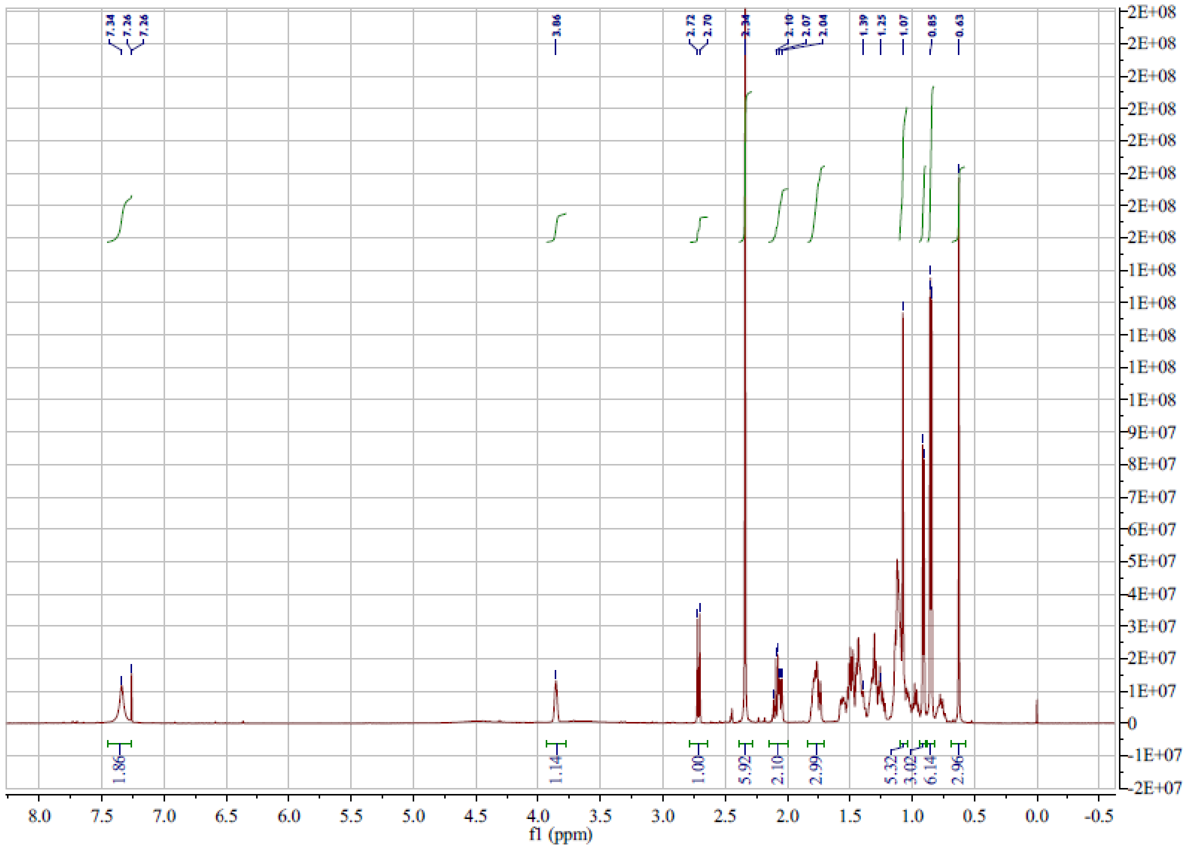

Figure S19. Compound **10a** ^{1}H NMR.

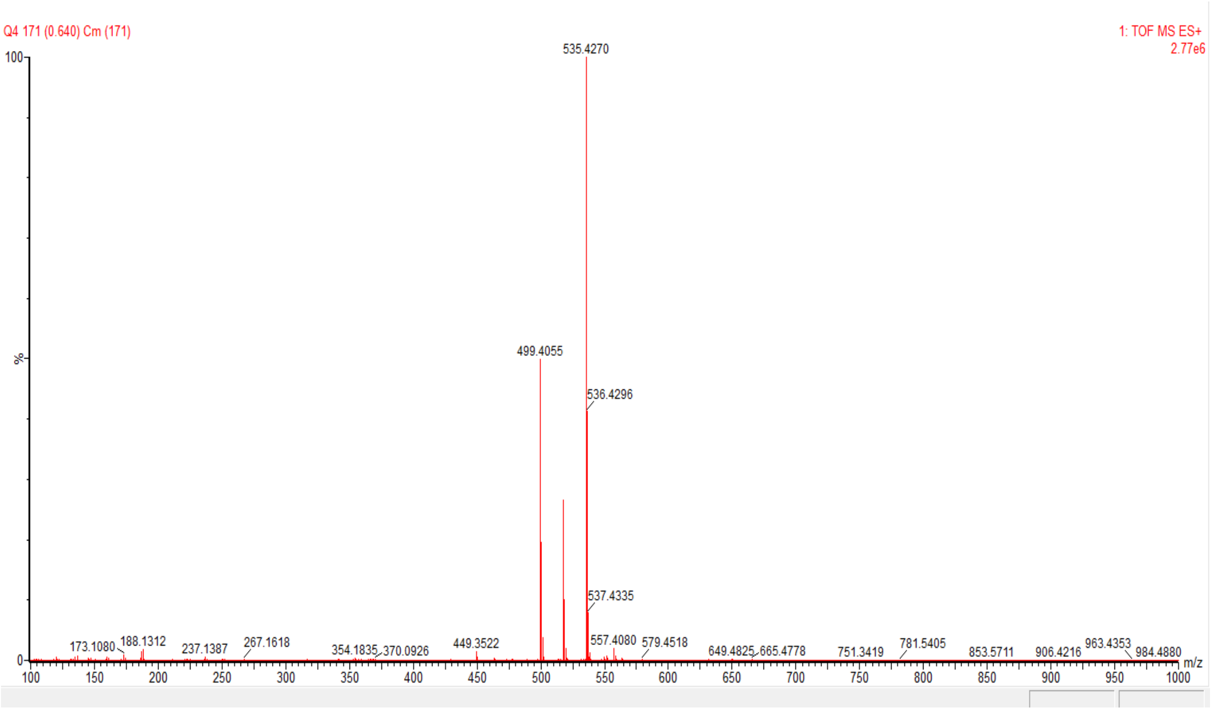

Figure S20. Compound **10a** HREIMS.

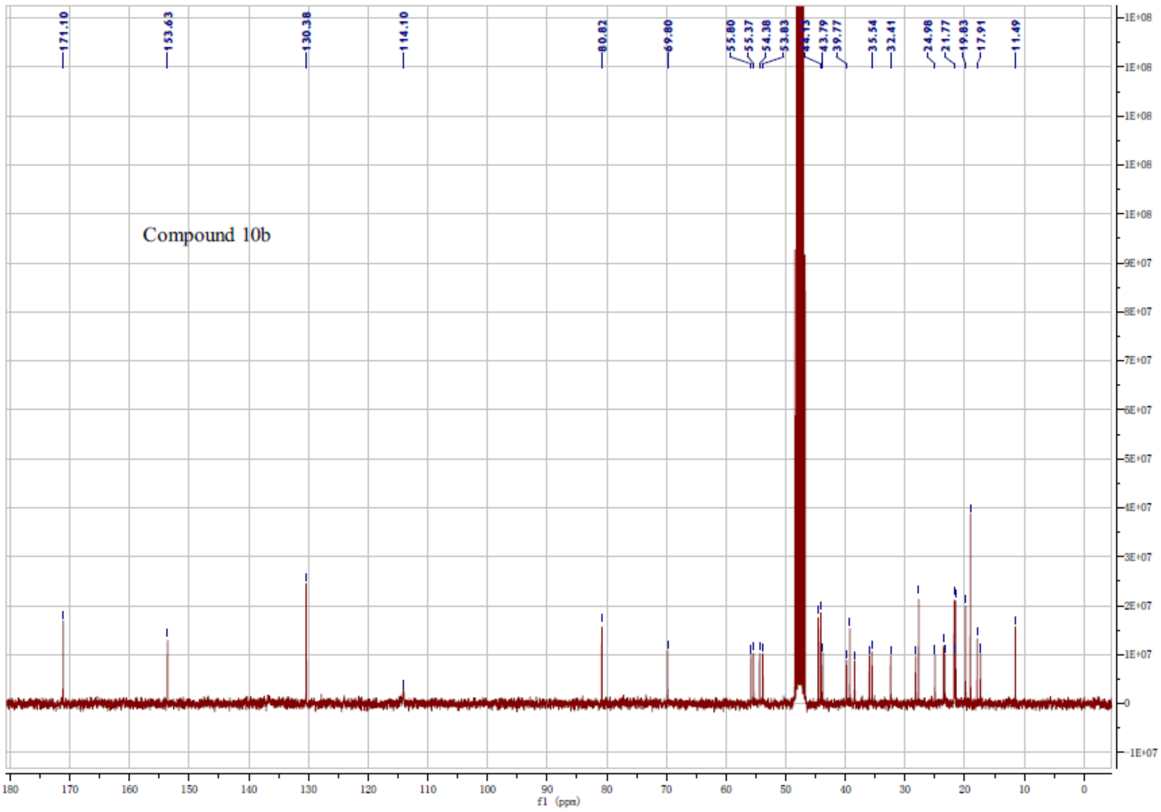

Figure S21. Compound **10b** ^{13}C NMR.

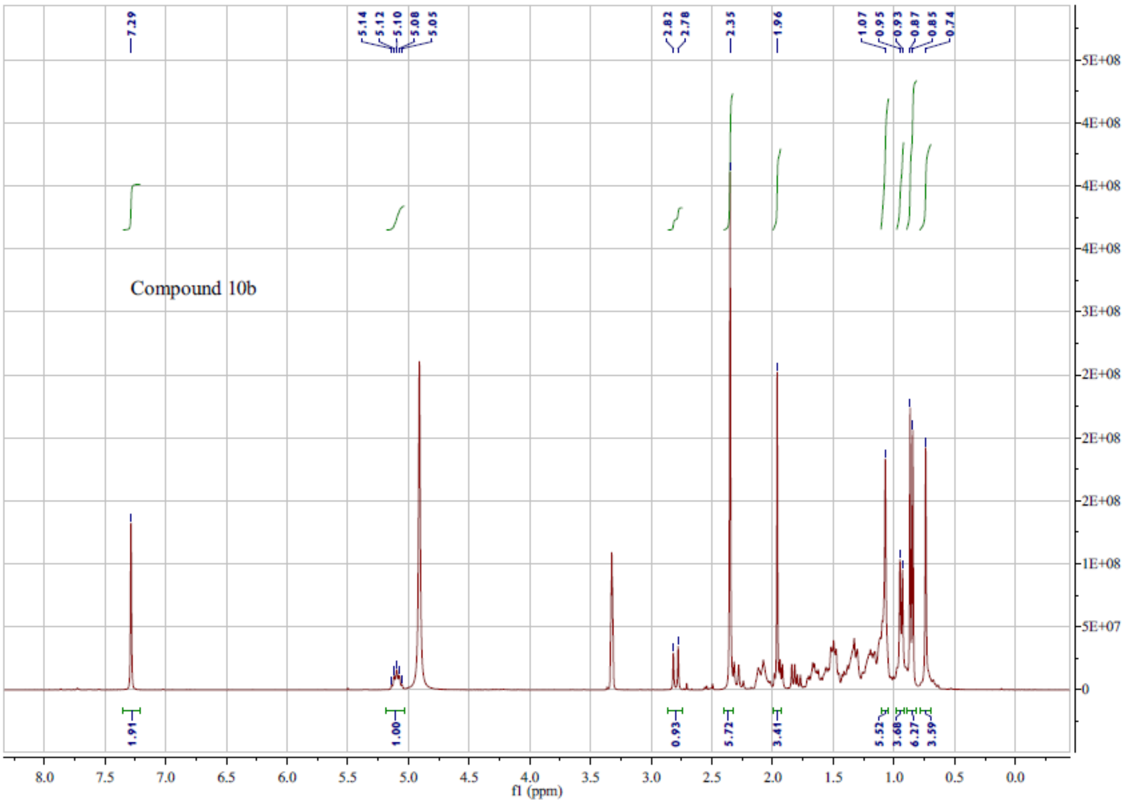

Figure S22. Compound **10b** ^{1}H NMR.

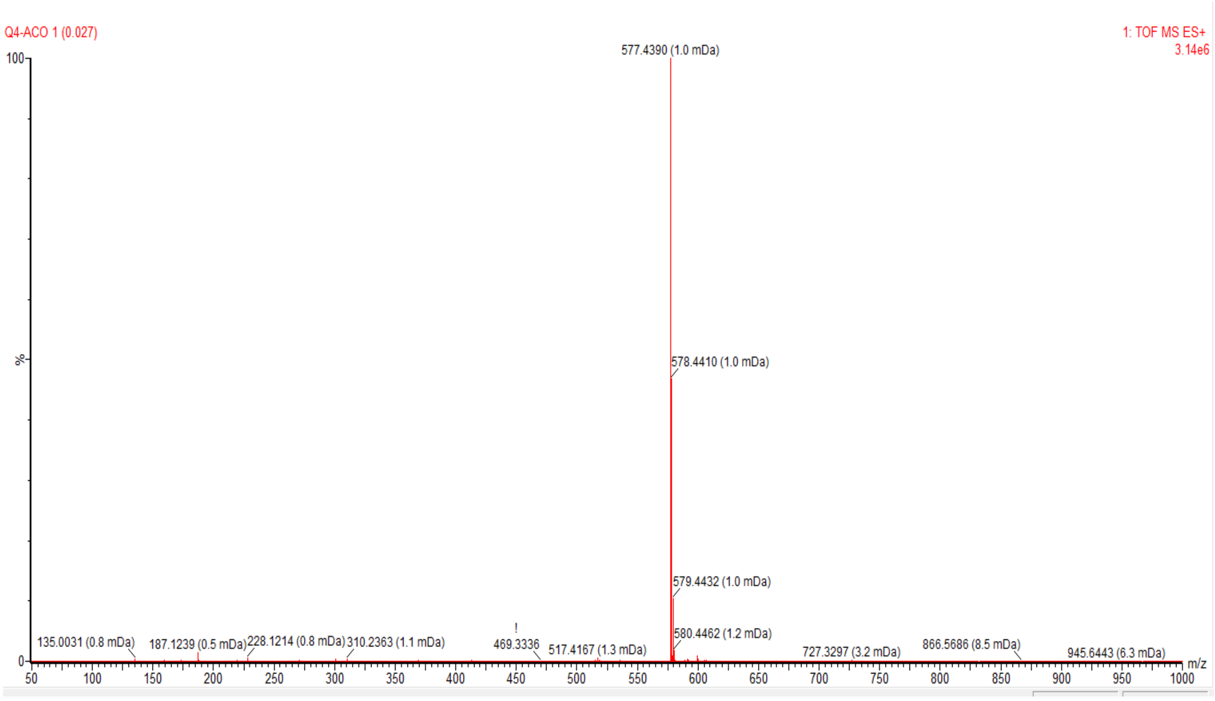

Figure S23. Compound **10b** HREIMS.

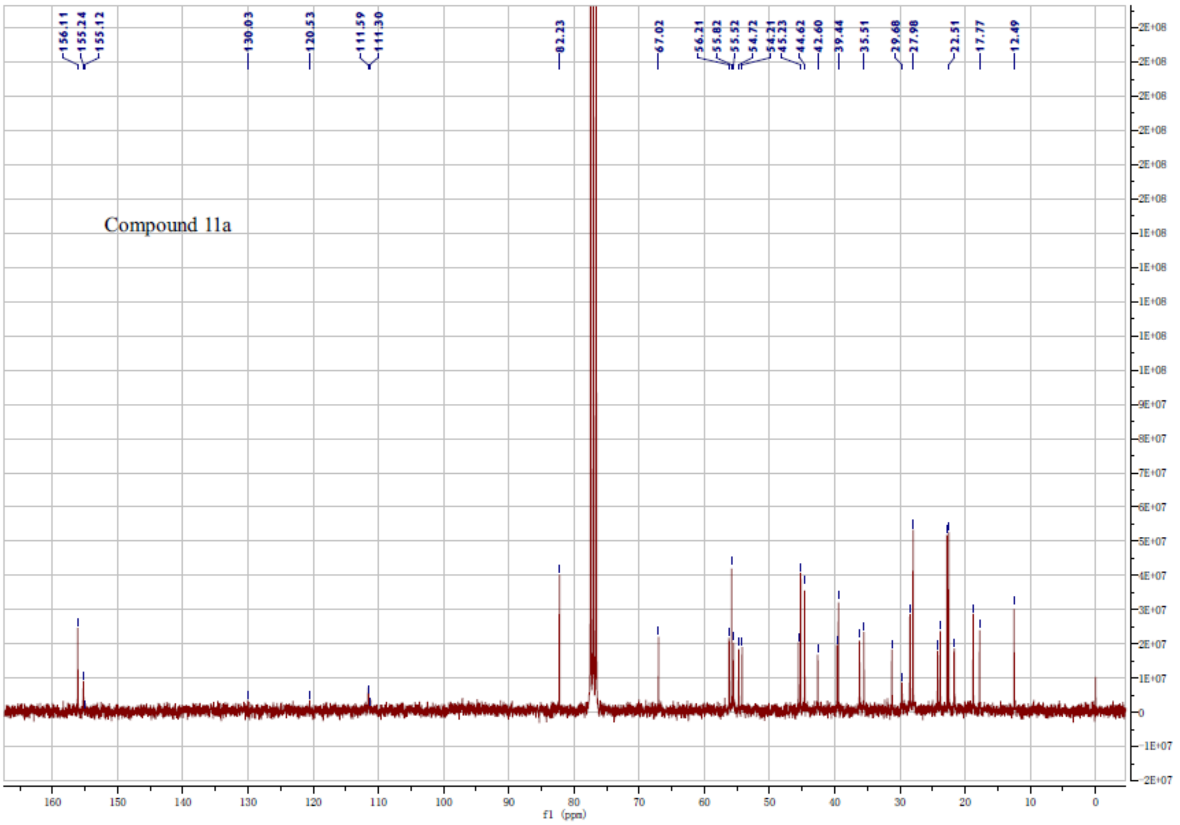

Figure S24. Compound **11a** ^{13}C NMR.

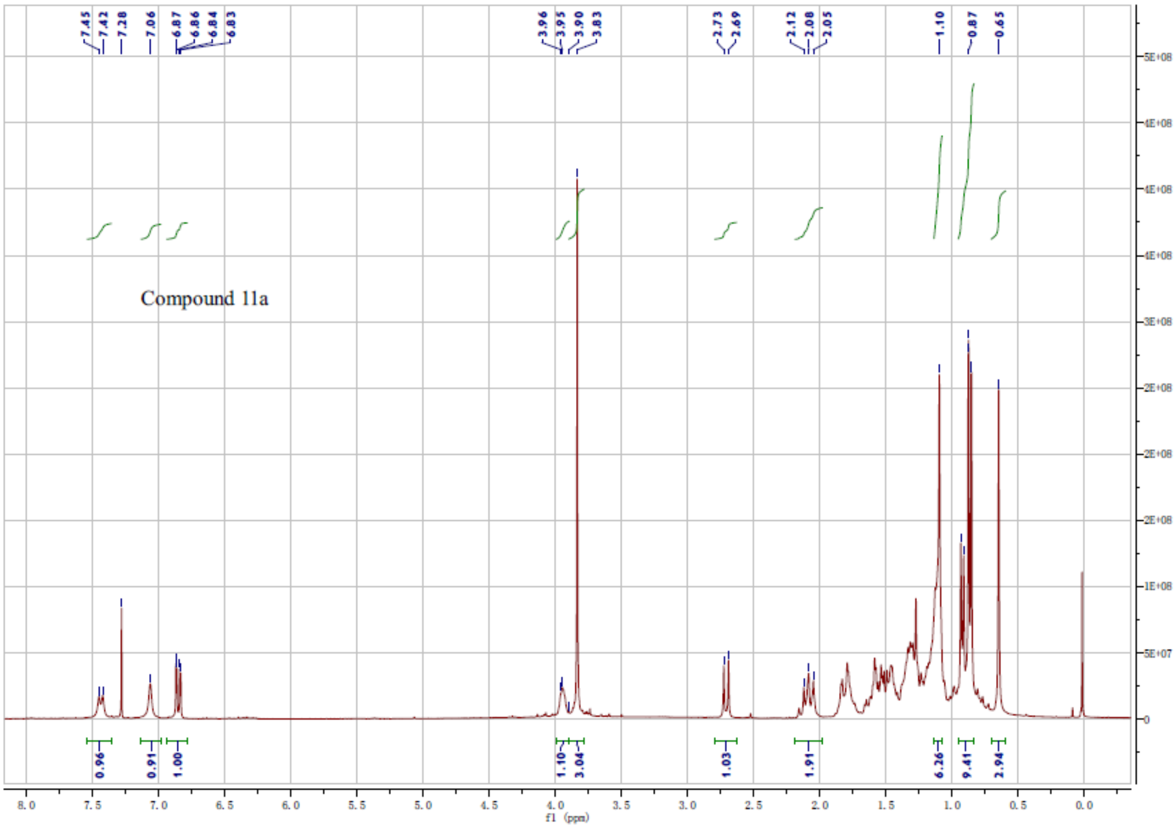

Figure S25. Compound **11a** ^{1}H NMR.

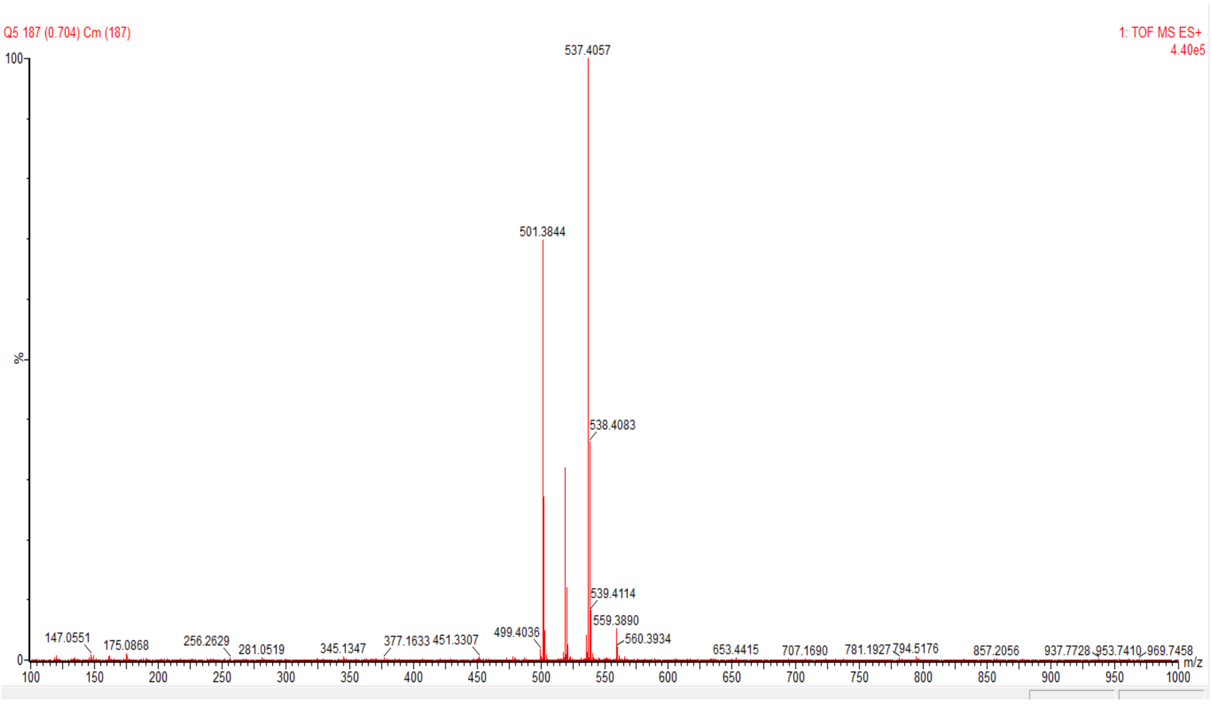

Figure S26. Compound **11a** HREIMS.

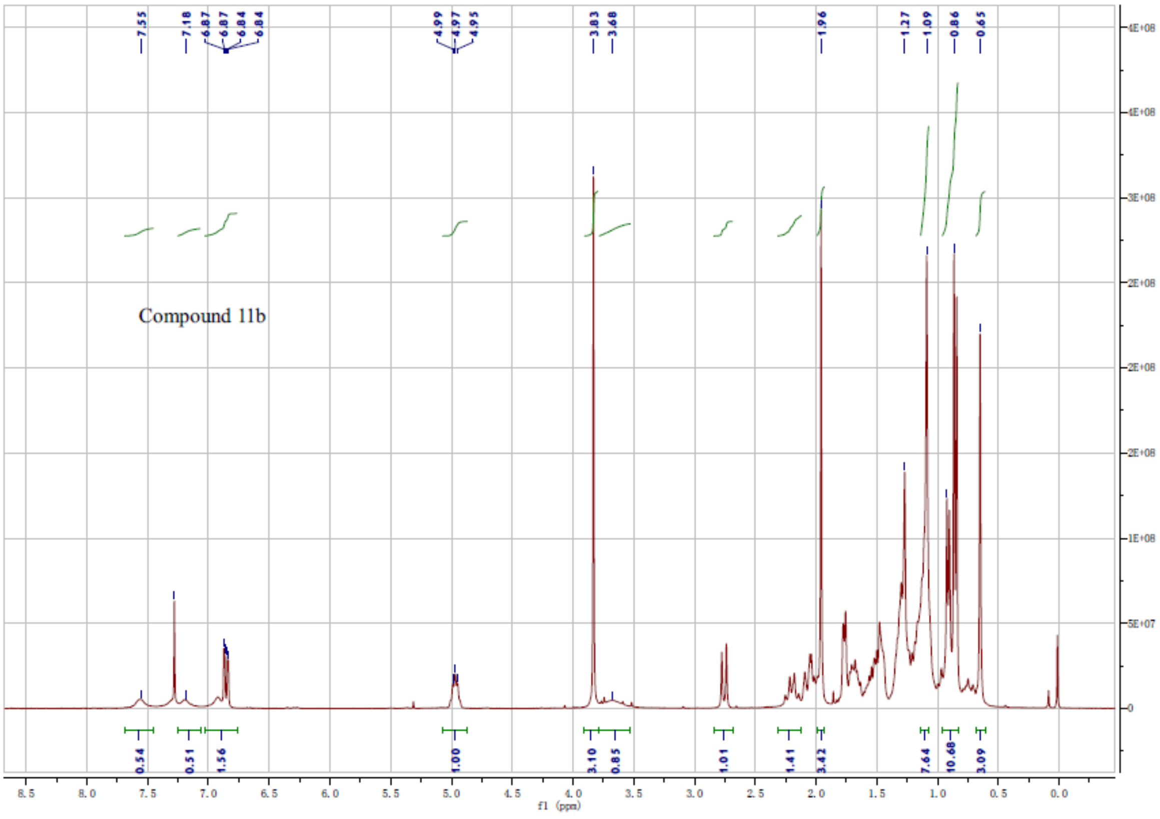

Figure S27. Compound **11b** ^{1}H NMR.

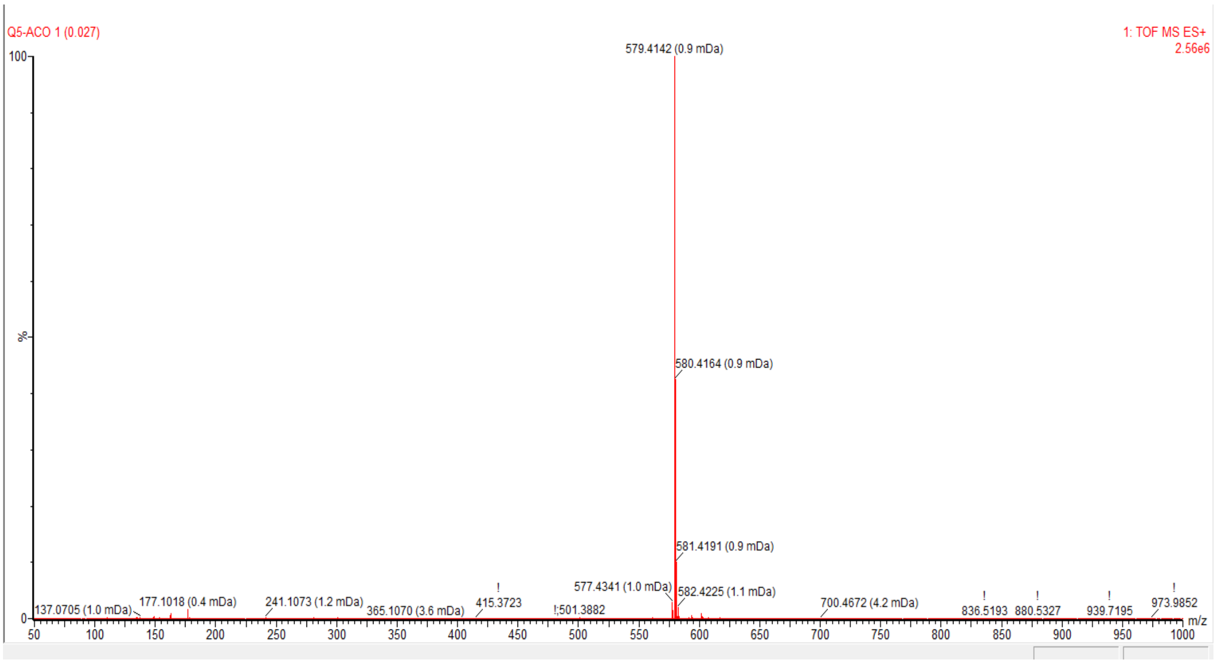

Figure S28. Compound **11b** HREIMS.

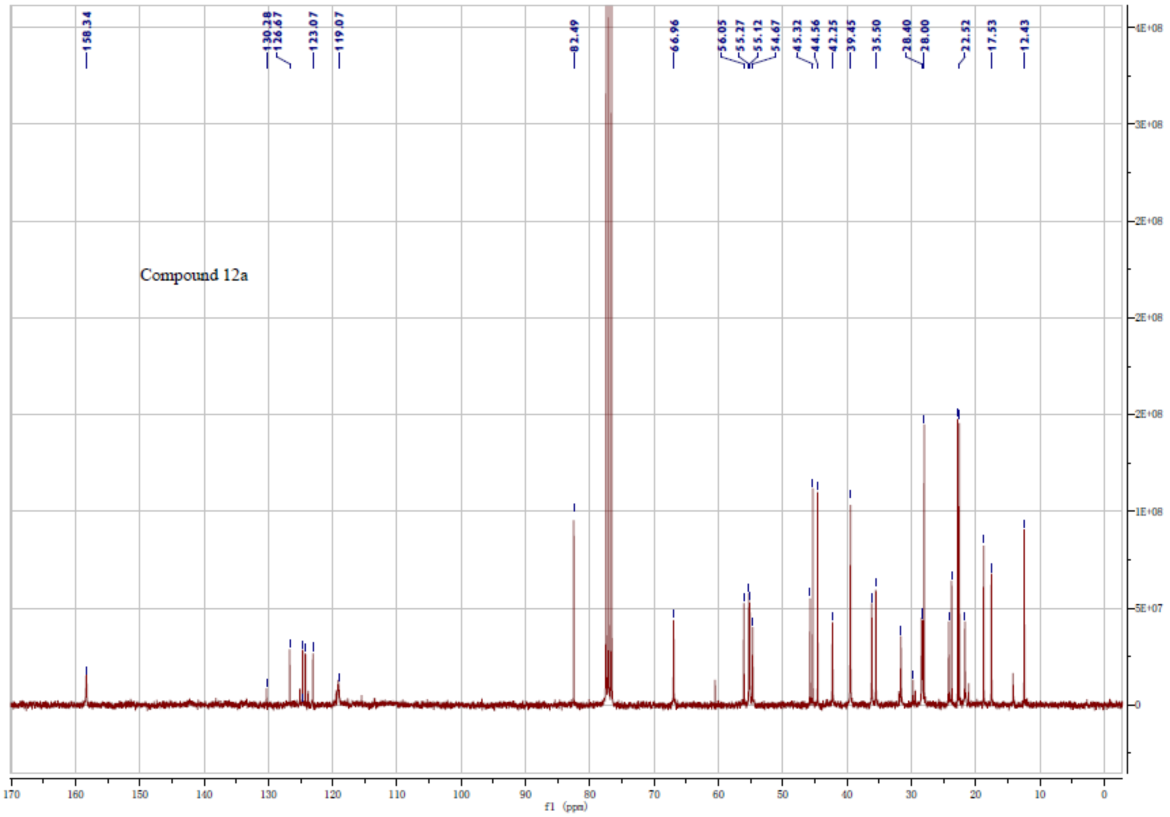

Figure S29. Compound **12a** ^{13}C NMR.

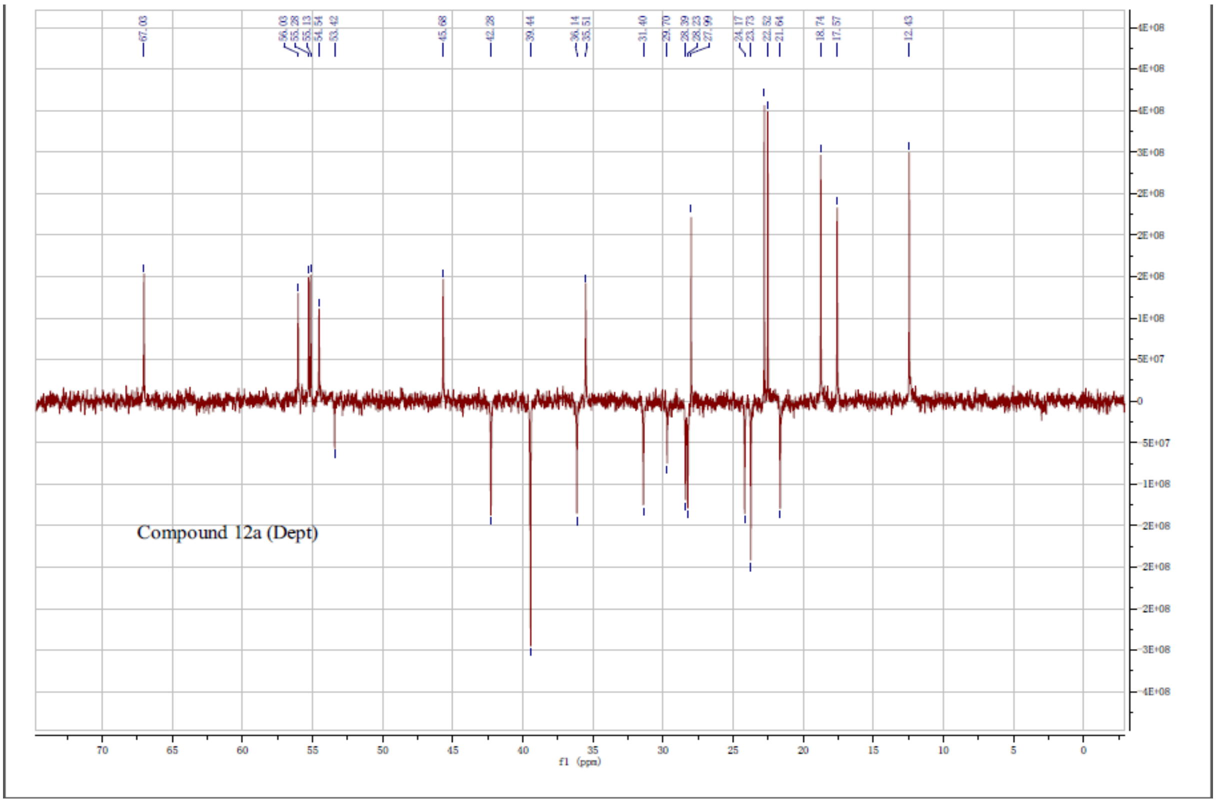

Figure S30. Compound **12a** ^{13}C NMR (Dept).

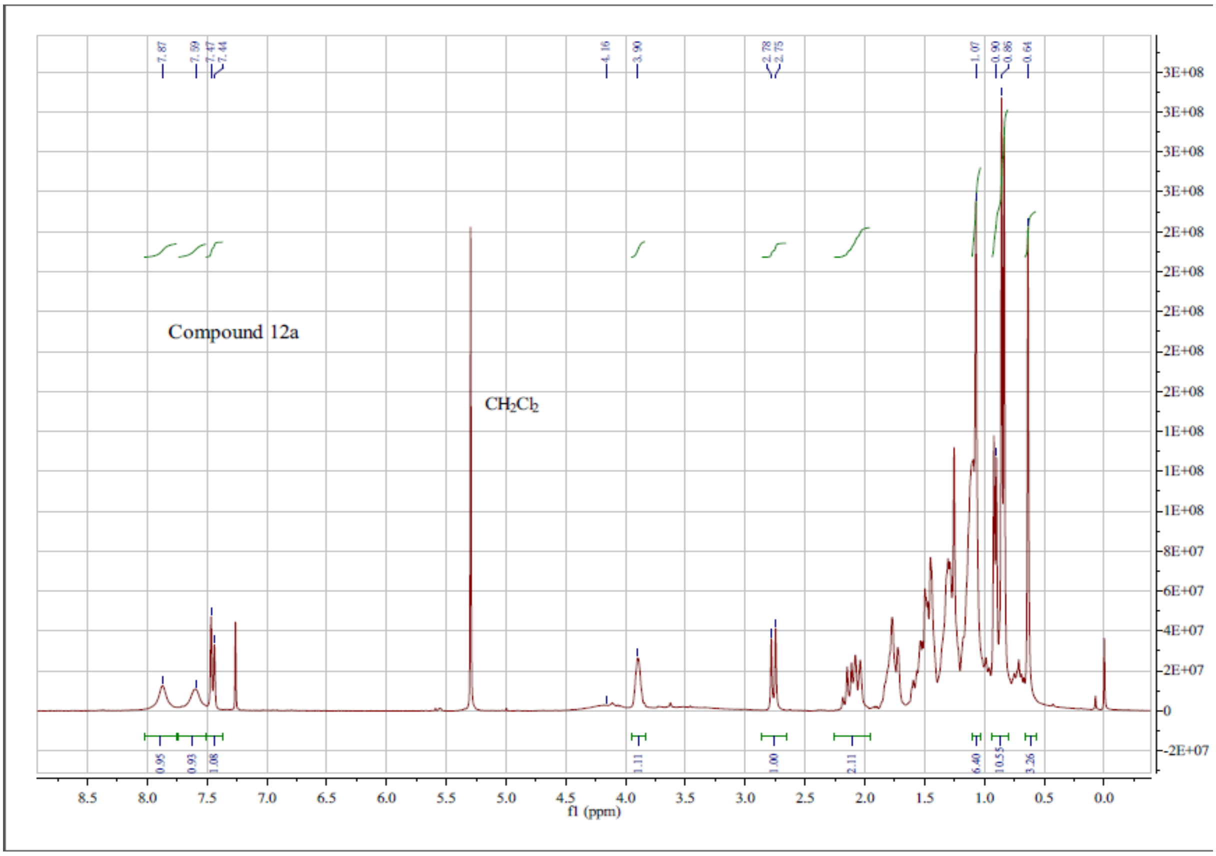

Figure S31. Compound **12a** ^{1}H NMR.

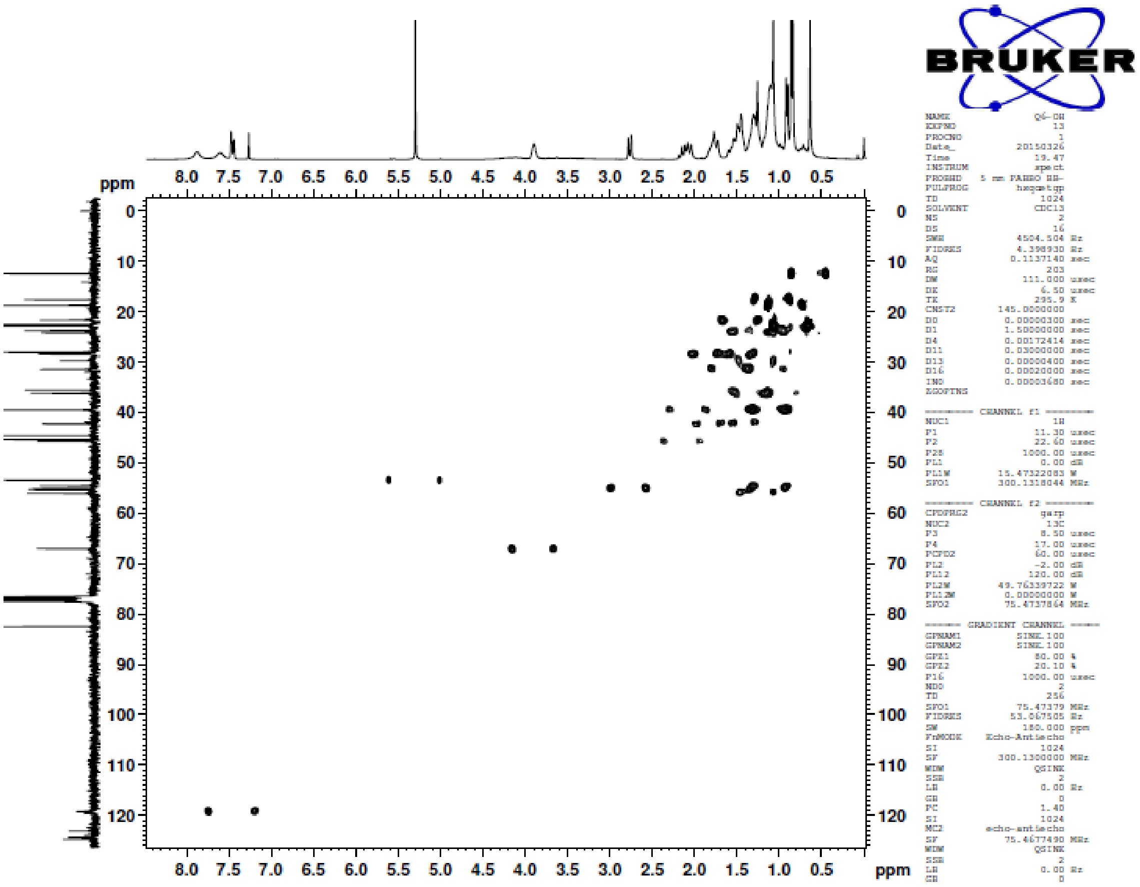

Figure S32. Compound **12a** HMQC.

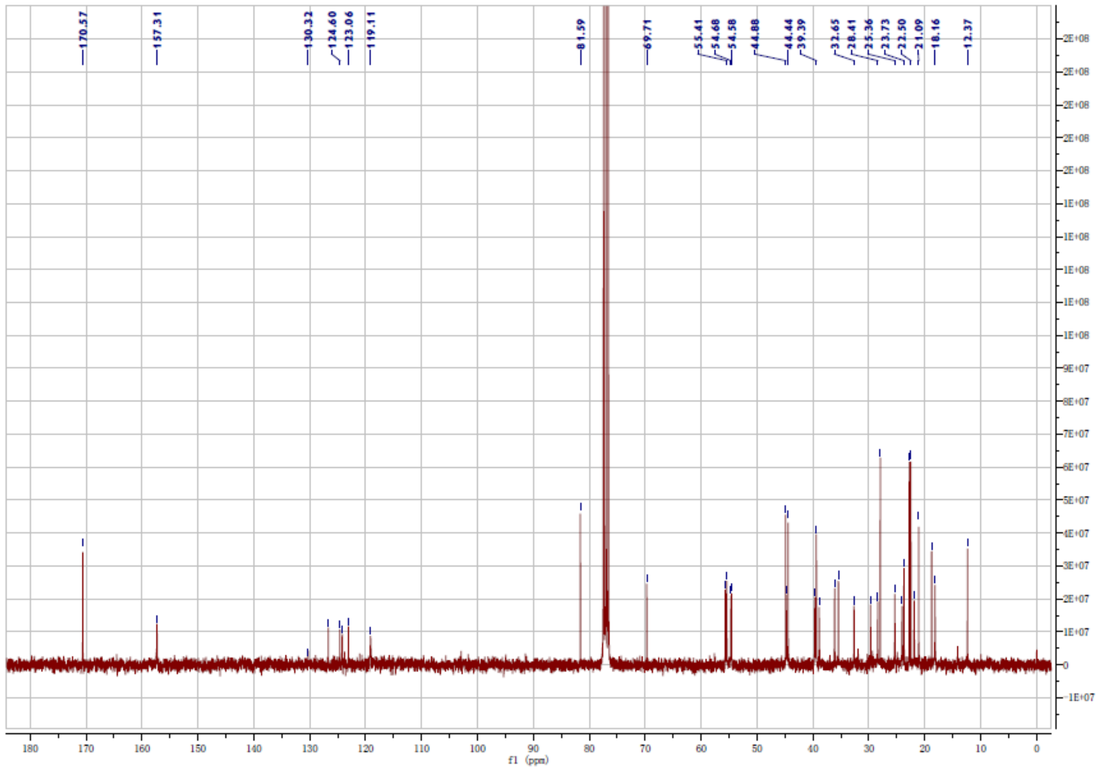

Figure S33. Compound **12b** ^{13}C NMR.

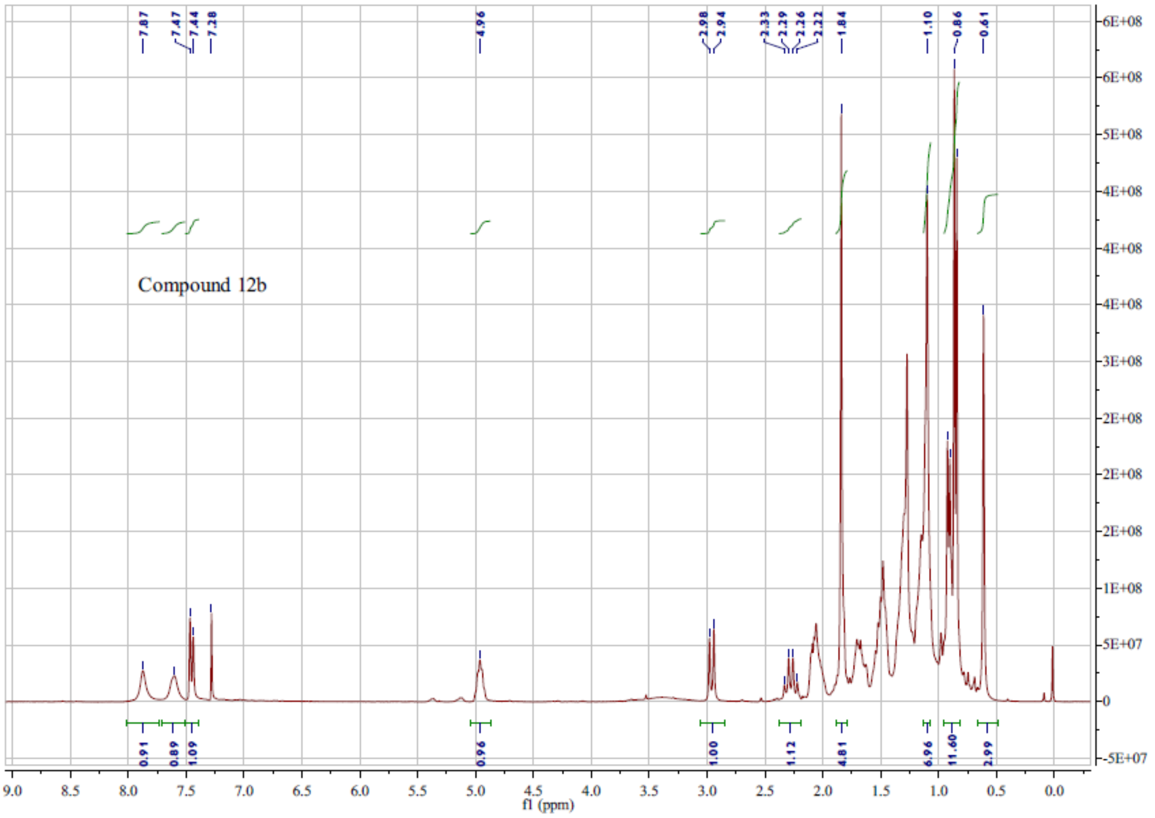

Figure S34. Compound **12b** ^{1}H NMR.

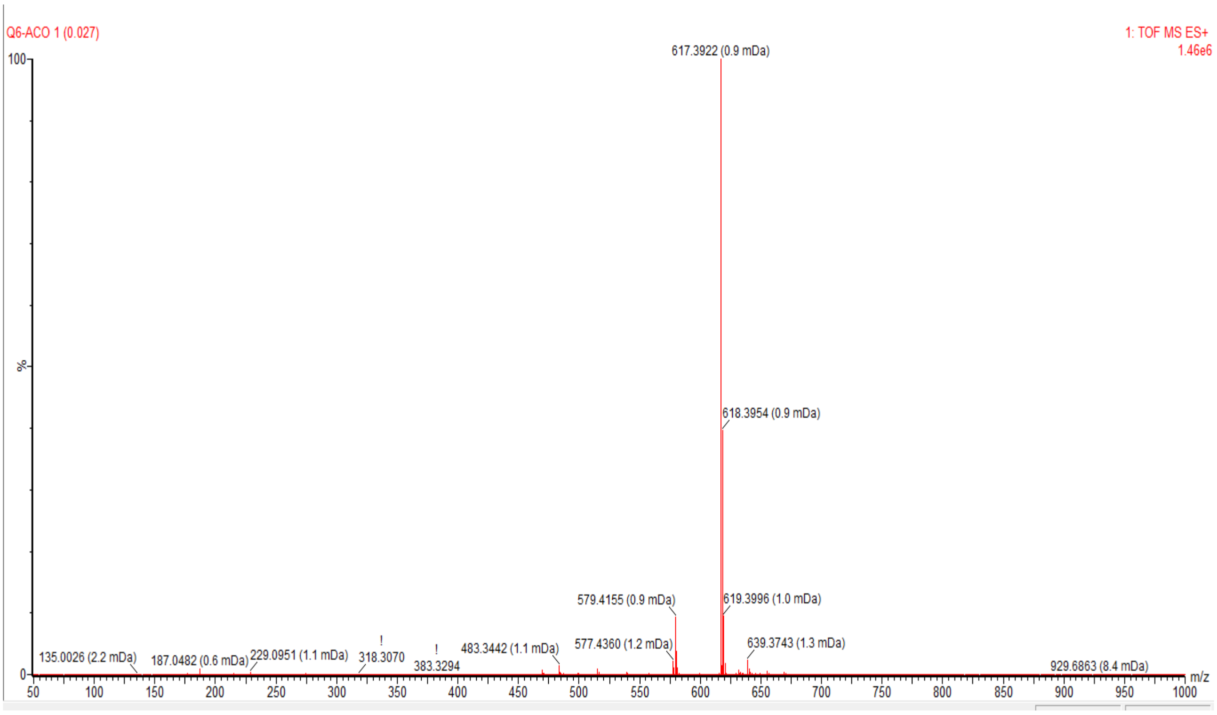

Figure S35. Compound **12b** HREIMS.

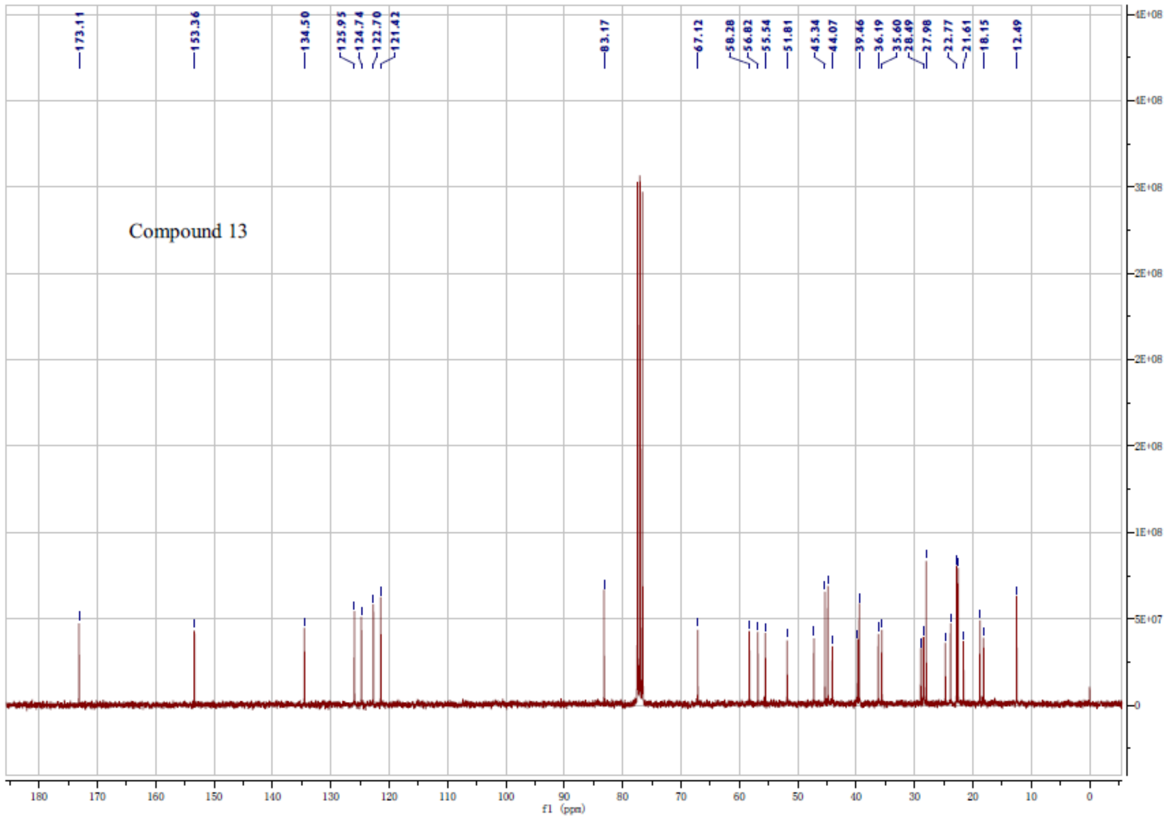

Figure S36. Compound **13** ^{13}C NMR.

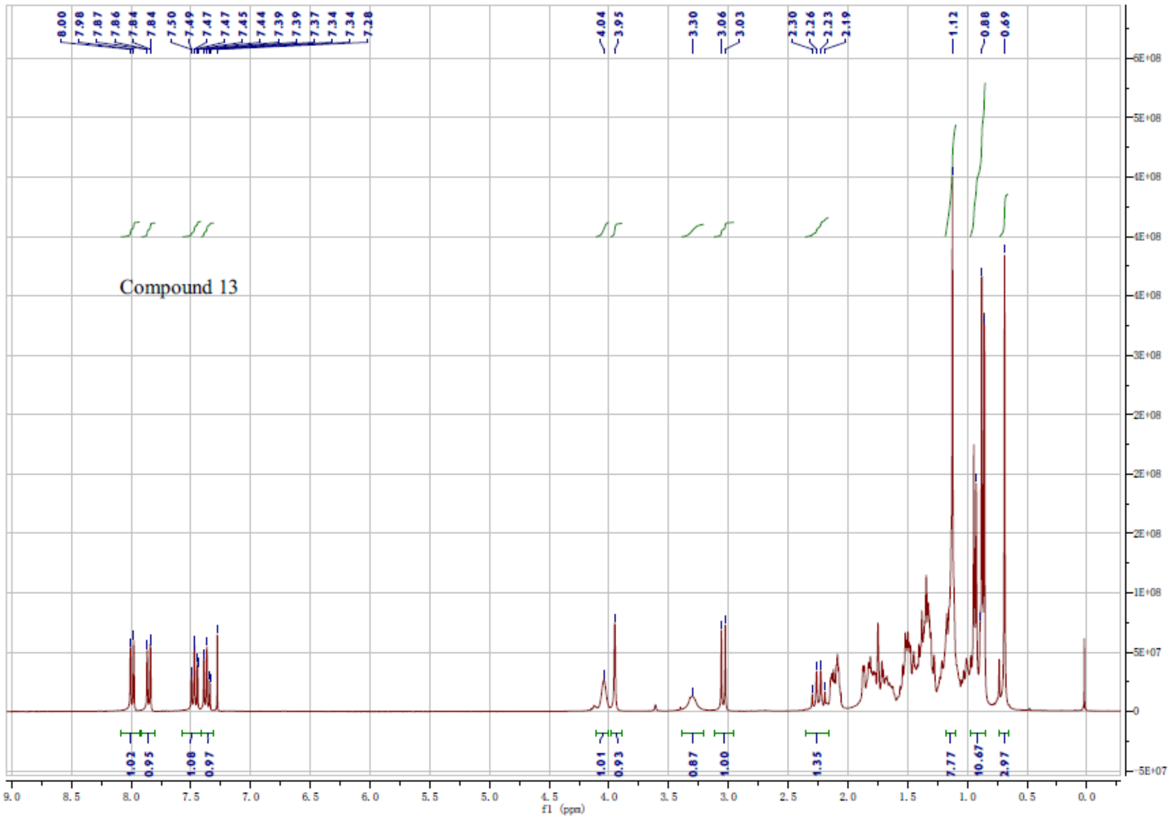

Figure S37. Compound **13** ^{1}H NMR.

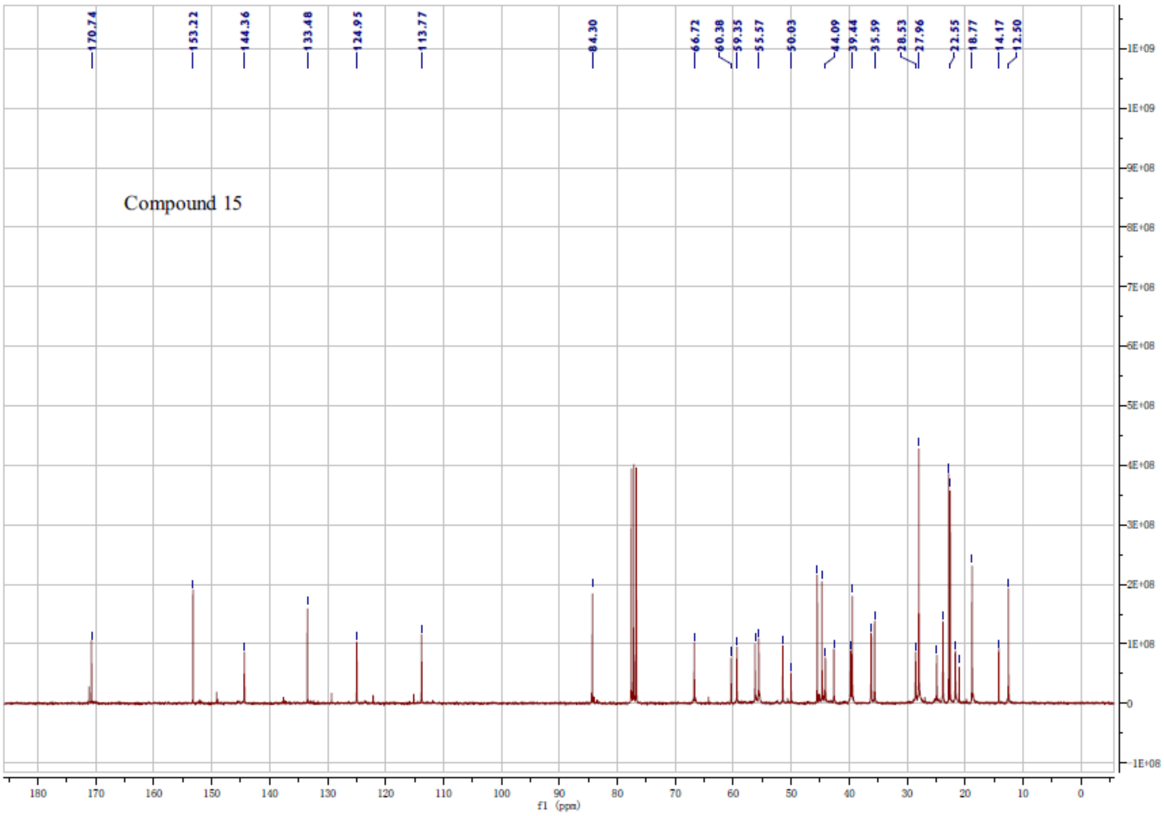

Figure S38. Compound **15** ^{13}C NMR.

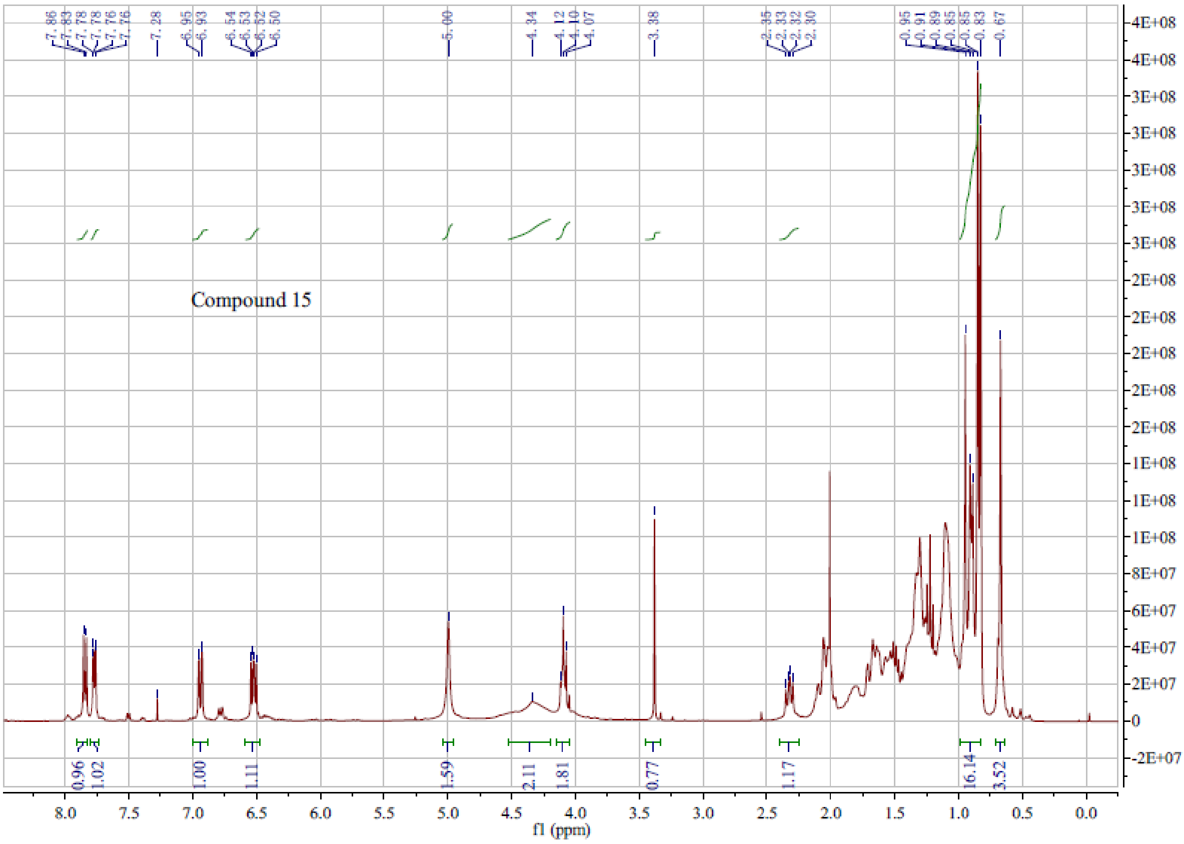

Figure S39. Compound **15** ^{1}H NMR.

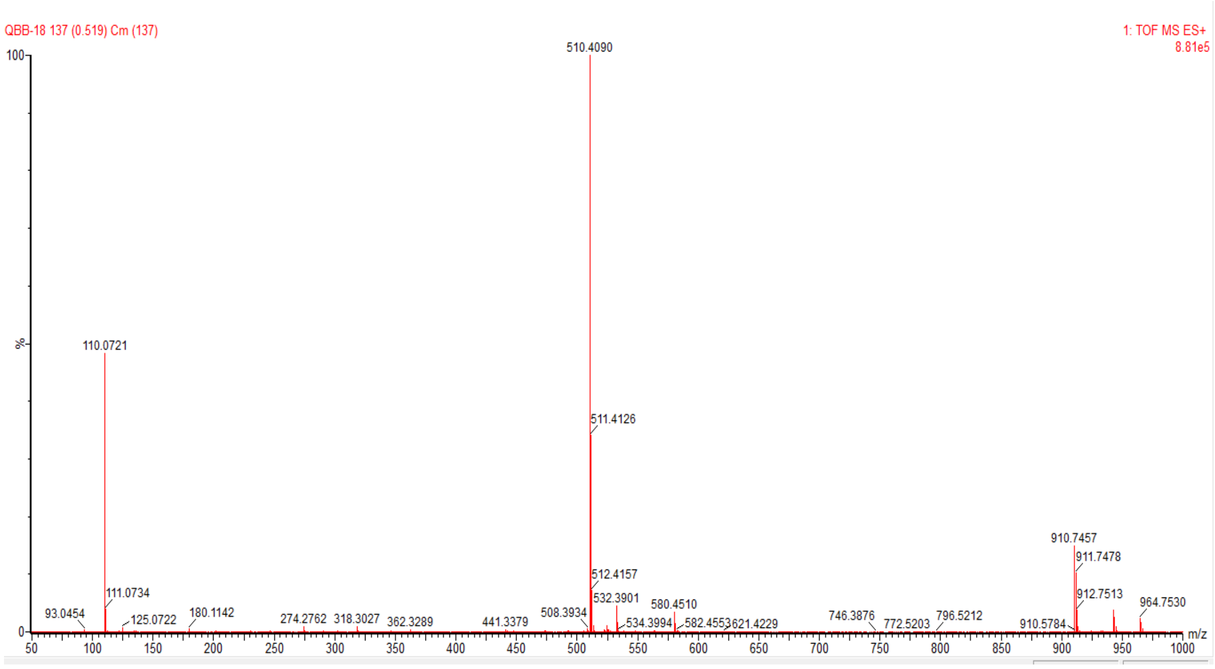

Figure S40. Compound **15** HREIMS.

© 2015 by the authors; licensee MDPI, Basel, Switzerland. This article is an open access article distributed under the terms and conditions of the Creative Commons Attribution license (http://creativecommons.org/licenses/by/4.0/).
